# Cancer-associated fibroblasts shape early myeloid cell response to chemotherapy-induced immunogenic signals in next generation tumor organoid cultures

**DOI:** 10.1136/jitc-2024-009494

**Published:** 2024-11-04

**Authors:** Julijan Kabiljo, Anna Theophil, Jakob Homola, Annalena F Renner, Nathalie Stürzenbecher, Daphni Ammon, Rebecca Zirnbauer, Simone Stang, Loan Tran, Johannes Laengle, Askin Kulu, Anna Chen, Markus Fabits, Velina S Atanasova, Oliver Pusch, Wolfgang Weninger, Henning Walczak, Dietmar Herndler Brandstetter, Gerda Egger, Helmut Dolznig, Anna Kusienicka, Matthias Farlik, Michael Bergmann

**Affiliations:** 1 Department of General Surgery, Division of Visceral Surgery, Medical University of Vienna, Vienna, Austria; 2 Ludwig Boltzmann Institute Applied Diagnostics, Medical University of Vienna, Vienna, Austria; 3 Comprehensive Cancer Center, Medical University of Vienna, Vienna, Austria; 4 Center of Pathobiochemistry and Genetics, Institute of Medical Genetics, Medical University of Vienna, Vienna, Austria; 5 Department of Pathology, Medical University of Vienna, Vienna, Austria; 6 Center for Cancer Research, Medical University of Vienna, Vienna, Austria; 7 Center for Anatomy and Cell Biology, Medical University of Vienna, Vienna, Austria; 8 Department of Dermatology, Medical University of Vienna, Vienna, Austria; 9 Institute for Biochemistry I, Medical Faculty, University of Cologne, Cologne, Germany; 10 Centre for Cell Death, Cancer, and Inflammation (CCCI), UCL Cancer Institute, University College London, London, UK

**Keywords:** Colorectal Cancer, Chemotherapy, Tumor Associated Macrophages, Cancer associated fibroblasts, Organoids

## Abstract

**Background:**

Patient-derived colorectal cancer (CRC) organoids (PDOs) solely consisting of malignant cells led to major advances in the understanding of cancer treatments. Yet, a major limitation is the absence of cells from the tumor microenvironment, thereby prohibiting potential investigation of treatment responses on immune and structural cells. Currently there are sparse reports describing the interaction of PDOs, cancer-associated fibroblasts (CAFs) and tumor-associated macrophages (TAMs) in complex primary co-culture assay systems.

**Methods:**

Primary PDOs and patient matched CAF cultures were generated from surgical resections. Co-culture systems of PDOs, CAFs and monocytic myeloid cells were set up to recapitulate features seen in patient tumors. Single-cell transcriptomics and flow cytometry was used to show effects of culture systems on TAM populations in the co-culture assays under chemotherapeutic and oncolytic viral treatment.

**Results:**

In contrast to co-cultures of tumor cells and monocytes, CAF/monocyte co-cultures and CAF/monocyte/tumor cell triple cultures resulted in a partial differentiation into macrophages and a phenotypic switch, characterized by the expression of major immunosuppressive markers comparable to TAMs in CRC. Oxaliplatin and 5-fluorouracil, the standard-of-care chemotherapy for CRC, induced polarization of macrophages to a pro-inflammatory phenotype comparable to the immunogenic effects of treatment with an oncolytic virus. Monitoring phagocytosis as a functional proxy to macrophage activation and subsequent onset of an immune response, revealed that chemotherapy-induced cell death, but not virus-mediated cell death, is necessary to induce phagocytosis of CRC cells. Moreover, CAFs enhanced the phagocytic activity in chemotherapy treated CRC triple cultures.

**Conclusions:**

Primary CAF-containing triple cultures successfully model TAM-like phenotypes *ex vivo* and allow the assessment of their functional and phenotypic changes in response to treatments following a precision medicine approach.

**WHAT IS ALREADY KNOWN ON THIS TOPIC**

- Patient-derived organoids (PDOs) of colorectal cancer recreate multiple features of cancer cells and enable scalable probing of treatment effects in cultures representing patient collectives. However, such cultures are missing essential cellular components of the tumor microenvironment, including cancer-associated fibroblasts (CAFs) and tumor-associated macrophages (TAMs). We have recently shown, that addition of CAFs in organotypic assays performed with PDOs leads to better recapitulation of cancer cell phenotypes compared with monoculture.

**WHAT THIS STUDY ADDS**

- We expanded on primary PDO-based organotypic assay systems to incorporate CAFs and monocyte-derived TAM-like cells. We show that contact between PDOs, CAFs and the myeloid compartment mutually maintains cancer-like differentiation and phenotype of all three cell types in the absence of artificial signals. On the other hand, organoid growth media containing chemical compounds and growth factors dramatically influenced the phenotype of TAM-like cells.

**HOW THIS STUDY MIGHT AFFECT RESEARCH, PRACTICE OR POLICY**

- Most studies using primary cultures of PDOs with immune cells apply organoid growth media during assays. Our study points to strong interference of such media with experimental outcomes in monocytic myeloid cells. The inclusion of CAFs can enable use of simple media as well, as induction of phenotypes similar to those observed in the tumor in PDOs and the myeloid compartment, creating a more physiologically relevant culture system. This will likely enhance clinical translation of results observed in such culture systems in the future.

## Background

 Monocytic myeloid cells are a highly plastic immune cell subset, capable of directing a wide variety of immune and tissue modeling responses depending on their activation state.^1^ As early responders to tissue injury or infection they direct both initial inflammation and post-inflammatory phases characterized by tissue-healing and fibrosis, making their functions in health and disease manifold. Cancers use various mechanisms to induce favorable phenotypes in myeloid cells, pushing this highly plastic cell type towards an immunoinhibitory phenotype often characterized and labeled as tumor-associated macrophages (TAMs).^2 3^ TAMs with immunoinhibitory features have also been linked to poor prognosis in colorectal cancer (CRC) cohorts, while detection of macrophage inflammation markers seemed to correlate with better prognosis.^4^ Thus, targeting TAMs and understanding their response to treatment will be relevant for the development of effective therapeutic strategies.^3 5^

Patient-derived tumor organoids (PDOs) appear exceptionally promising to investigate the interaction between TAMs or TAM-like cells and cancer cells of individual patients *ex vivo*, in a sufficiently scalable manner.^6^ At the same time, the field of macrophage biology has been hampered by conflicting results originating from mouse models, making a case for the development of preclinical human models to better understand the role of myeloid cells in the human tumor microenvironment (TME).^7^ PDOs were developed during the past decade and have major advantages compared with traditional cell line systems, specifically a high similarity to human cancer as it presents *in vivo*, including preserved cellular heterogeneity^8^ and highly stable epigenetic profiles.^9^ While such PDOs have enabled predicting responses to certain chemotherapeutics as well as radiotherapy, responses to a number of standard-of-care therapeutics were not well recapitulated.^10^,^13^ This is likely due to the fact that these PDOs lack cells contributing to the TME, especially immune cells within the experimental set-ups. Some studies have attempted to supplement cells from the immune compartment, investigating T-cell co-culture with PDOs.^14 15^ Studies exploring the interaction of CRC-PDOs and immune cells have so far often disregarded other elements of the TME such as cancer-associated fibroblasts (CAFs).^16 17^ We have recently shown, that CAFs are essential for the maintenance of PDO differentiation and a transcriptomic profile similar to phenotypes seen in the TME, also allowing for the omission of multiple additives in the PDO culture medium, which are necessary for PDO growth in monoculture, but are likely to interfere with multiple assay readouts.^18^ We therefore hypothesize that CAFs are similarly essential for modeling treatment responses of TAM-like cells in a reconstituted primary CRC culture set-up.

Here we generated *ex vivo* organotypic co-culture assays to study the effects of treatments on monocyte-derived myeloid cells in CRC, reconstituting essential components of the complex microenvironment through the addition of CAFs. An oncolytic influenza A virus (O-IAV), previously generated in our laboratory,^19 20^ served as a model treatment with strong expected immunostimulatory effects on macrophages. We compared virotherapy to standard-of-care chemotherapeutics, oxaliplatin and 5-fluorouracil (5-FU), which are only loosely associated with anticancer immunity and appear to have both immuno-stimulatory and immuno-suppressive properties.^21^,^23^ Importantly, due to practical and ethical restrictions, we only have very limited knowledge of chemotherapy-induced changes in monocytic myeloid cells during the acute phase within the first 24 hours after treatment. Our approach identified differential responses of monocytes and macrophages in the co-culture with primary CAFs. We revealed a strict functional dependency of macrophage phagocytosis on the presence of CAFs in the triple-culture system and characterized unexpected immunostimulatory properties of chemotherapy-induced cell death on both TAM-like cells and CAFs. Our findings re-define the role of monocyte-derived macrophages and CAFs in the TME and underline the utility of the here established culture systems for precision medicine and drug screening approaches.

## Methods

### Cell lines, viruses and chemotherapeutics

Vero African green monkey kidney cells were cultured in OptiPro SFM and were purchased from the American Type Culture Collection (ATCC).

O-IAV-green fluorescent protein (GFP) and O-IAV viruses were generated based on H1N1-NS1_116_-GFP previously generated in our laboratory,^20^ with O-IAV viruses expressing the A/Chicken/Kurgan/05/2005 hemagglutinin (GenBank Accession: DQ449632) instead of A/Puerto Rico/8/34 hemagglutinin. The avian polybasic cleavage site was modified to a trypsin site (TETR/GLF), reversing pathogenicity.^24^ Titration of viruses was performed using a tissue culture infective dose 50% (TCID_50_) assay.

Oxaliplatin and 5-FU were kindly provided by the internal pharmacy of the Vienna General Hospital.

Catalog numbers and manufacturers of all materials used in this study are available in the online supplemental table 1.

### Isolation of primary monocytes and macrophages

Whole blood of young, healthy, age and sex matched volunteers as well as patients with CRC was collected in VACUETTE EDTA tubes. Monocytes were isolated using the EasySep Direct Human Monocyte Isolation Kit.

For macrophage generation monocytes were attached to cell culture plates for 2 hours in Roswell Park Memorial Institute (RPMI)-1640. Adherent monocytes were further incubated in RPMI-1640 supplemented with 10% fetal calf serum and 100 ng/mL macrophage colony stimulating factor (M-CSF) for 7 days, yielding macrophages.

### Primary fibroblast culture

CAFs were cultured as previously described.^18^

### Generation of primary CRC organoids

CRC organoids were cultured as previously described.^6 18^ The source material for organoid generation was CRC tissue dissected from the colon or metastatic liver obtained intraoperatively from the resected tissue. Tissue was cooled on ice for less than 15 min until dissection. Patients were stable during the operation. The pathological diagnosis was colon adenocarcinoma. Tissues were subjected to freezing in a Corning CoolCell system at −80°C in CELLBANKER-2 (ASMBIO) overnight and further stored in liquid N2 for 1–12 months until processing for organoid and fibroblast culture. Samples were included in further experiments if organoid growth was achieved.

### Infection of organoids

For Geltrex submerged infection of organoids, domes containing organoids were incubated with 5×10^2^ TCID_50_/initially seeded cell for 1 hour at 37°C in a 5% CO_2_ atmosphere under slight agitation and subsequently incubated in culture medium. Domes were subjected to dissociation by 30 min incubation in 2.5 U/mL dispase. Single-cell suspension was achieved by incubation in TrypLE. Readout of infectivity was performed by flow cytometry for cells expressing the viral GFP tag.

For quantification of viral titers, organoids were infected in a Geltrex submerged culture as described above and incubated for 24 hours in the presence of 1 µg/mL L-1-tosylamide-2-phenylethyl chloromethyl ketone-treated trypsin. Supernatants were collected and infective virus titer was quantified by TCID_50_.

### PDO viability

PDOs were seeded on day 0 and subjected to media change on days 2, 4 and 5. Treatments were started with seeding, on day 4, on day 5, or completely omitted for untreated control conditions. For O-IAV, cultures were infected using 5×10^2^ TCID_50_/initially seeded cell for 1 hour and subsequently incubated in ENAS. For chemotherapy, cultures were incubated in ENAS in the presence and absence of 20 µM oxaliplatin and/or 0.5 mM 5-FU. Cell viability was determined by CellTiter-Glo 3D according to the manufacturer’s instructions. Luminescence was determined using Varioskan LUX.

### Triple culture

Fibroblast-conditioned medium was generated by a 3-day low-volume culture of CAFs in 4-(2-hydroxyethyl)-1-piperazineethanesulfonic acid (HEPES) buffered advanced DMEM/F12 containing GlutaMAX (1×) and 1% fetal bovine serum (FBS) (basal medium). Primary monocytes from volunteer donors and CAFs were detached using TrypLE and organoids were released from Geltrex using dispase. The cells were embedded in Geltrex domes at a ratio of 20:10:1 (CAFs:monocytes:organoid-cells) for all triple culture assays (for some controls one or more cell types were omitted while keeping the same cell number per culture; if cell types are omitted, detailed description of present cell types is indicated within the figures underneath the subplot). For the set-up, the domes were cultured in basal medium, fibroblast conditioned medium or ENAS for 72 hours. For infection, domes were cultured in basal medium for 3 days, infected using 5×10^2^ TCID_50_/initially seeded cell for 1 hour and cultured in basal medium for a further 24 hours. For chemotherapy, domes were incubated in a basal medium containing 20 µM oxaliplatin and/or 0.5 mM 5-FU for 24 hours. All domes were then subjected to dispase and TrypLE incubation and the resulting single-cell suspension was either subjected to single-cell transcriptomics as described below or stained with fluorescence-labeled antibodies (online supplemental table 2) and analyzed by flow cytometry.

### Triple culture readouts

For H&E and immunofluorescence (IF) stainings extracellular matrix (ECM) domes were fixed in 1% paraformaldehyde for 1 hour and embedded in agarose. Agarose-embedded domes were fixed a second time in 1% paraformaldehyde for 1 hour, dehydrated and embedded in paraffin. 3 µm sections were applied on SuperFrost Plus slides. Slides were heated to 56°C for 12 hours and subsequently rehydrated. H&E staining was performed as previously described.^25^ For IF, slides were subjected to antigen retrieval at 121°C under high pressure in 10 mM sodium citrate with 0.05% Tween 20 at pH=6. Slides were washed with TRIS-buffered saline solution (TBS), permeabilized in 0.2% Triton X-100 for 5 min, washed again in TBS with 0.05% Tween 20 (TBS-T), and blocked with 10% goat serum, 1% bovine serum albumin, 0.05% Tween 20 and 0.3M Glycin for 60 min. Primary staining was performed using an anti-vimentin antibody at 1:200 dilution and an anti-CD45 antibody at 1:100 dilution at 4°C in a humid chamber overnight. Slides were washed with TBS-T and incubated with secondary antibodies against pan-keratin at 1:100 concentration and Alexa Fluor 546 conjugated anti-rabbit IgG at 1:400 concentration as well, as Alexa Fluor 647 conjugated anti-mouse IgG at 1:400 concentration. Slides were washed with TBS-T and finally stained with 4’,6-diamidino-2-phenylindole, dihydrochloride (DAPI) at 1:1,000 dilution before a final wash with TBS-T and mounting in Fluoromount-G. Slides were imaged as described below.

Supernatants from treated cultures were shock-frozen. Aliquots were thawed and subjected to LEGENDplex analysis according to the manufacturer’s instructions. A customized kit measuring interleukin-6 (IL-6), IL-1β, tumour necrosis factor (TNF) and IL-10 was used. The assay was acquired using the DxFlex flow cytometer. Flow cytometry data was converted to cytokine concentrations by use of LEGENDplex analysis software according to the manufacturer’s instructions.

### Sample preparation for single-cell RNA-sequencing

For the CAF-monocyte/macrophage experiment co-cultures at a 2:1 CAF:monocytic cell ratio and single cultures of CAFs or macrophages with equivalent seeding densities were cultured for 24 hours in basal medium. Cells were subsequently counted and untreated CAFs pooled with untreated macrophages or freshly isolated monocytes, and further processed using the 10x Genomics workflow as described below.

Triple cultures were set up and treated as described above. Single cells were stained with DAPI and live DAPI-negative cells were sort purified and used for single-cell sequencing.

### Single-cell RNA-sequencing

Single-cell RNA-sequencing (scRNA-seq) was performed using the 10x Genomics Chromium Next GEM 3’ v3.1 (CAF+mono/macro experiment) or 5’ v2 (triple culture experiment) kit and run on a Chromium Single Cell Controller. Complementary DNA recovery, amplification and gene expression libraries were prepared according to the manufacturer’s protocol. Quality control was performed using TapeStation (Agilent) and Qubit Fluorometer (Thermo Fisher). Libraries were sequenced on a NovaSeq SP flow cell (Illumina) with 50 bp read length in paired-end mode.

The scRNA-seq data set was processed, explored and visualized using the Trailmaker (https://app.trailmaker.parsebiosciences.com/) pipeline (Parse Biosciences, formerly Biomage-hosted community instance of Cellenics). Count matrices for individual experiments (CAFs+mono/macro and triple culture) were separately uploaded onto Trailmaker. Employing the distribution of mitochondrial read percentages in each sample, a threshold was established at two median absolute deviations above the median. Cells surpassing this threshold in mitochondrial read percentages were eliminated (mitochondrial content cut-off range: 6.8–12%). This step led to the removal of 2.75–8.65% of cells, depending on the sample. The *rlm* method of the MASS R package (V.7.3–56) was used to adjust a robust linear model to the relationship between the number of genes with at least one count and the number of uni-molecular identifiers (UMIs) of each droplet.^26^ A fitted model (with a tolerance level of 1 − alpha, where alpha is one divided by the number of droplets in each sample) was used to predict the number of genes for each droplet and droplets outside the boundaries of the prediction interval were filtered out (alpha value cut-off range: 8.40E-05–6.4E-04, 0.1–0.74% of cells filtered out). The doublets rate was calculated for each sample using the scDblFinder R package (V.1.11.3)^27^ leading to the removal of 6.06–13.71% of cells. Sample-specific thresholds for all samples are reported in online supplemental table 3. After processing, 1,458–10,334 high-quality cells were kept with overall filtering rates of 12.87–17.07%. The data set underwent log-normalization, followed by the selection of the top 2,000 highly variable genes based on the variance stabilizing transformation method. Subsequently, principal component analysis was conducted, using the top 25 principal components (PCs) for the CAF+mono/macro experiment, 22 PCs for the triple culture experiment and 15 PCs for the re-clustered CAFs from the CAF+mono experiment. These components collectively explained 76–90% of the total variance of experiments. Integration of these components was performed using the Harmony R package.^28^ Clustering was carried out via Seurat’s implementation of the Louvain method, and cluster-specific marker genes were identified by comparing cells within each cluster against all other cells using the *presto* package implementation of the Wilcoxon rank-sum test.^29^ Finally, visualization of the results was achieved through a Uniform Manifold Approximation and Projection (UMAP) embedding.

### Differential expression analysis

Differential expression analysis was performed using Trailmaker analysis tool and batch differential expression method. Within a selected cell type, different samples were compared with respective control samples. The resulting tables with deregulated genes (DE) are included in online supplemental table 4. Genes were sorted based on the logFC values (descending for upregulated and ascending for downregulated genes). For further analysis, top 25/100/200 genes with equal or more than 0.5 average expression values were selected, and all the non-coding RNAs and pseudogenes were filtered out. The resulting list of genes was used for the Gene Set Enrichment Analysis (GSEA) using the Enrichr tool.^30^,^32^

### Flow cytometry and microscopy

All cells were incubated in 40% human AB serum for 5 min before staining and subsequently stained for 30 min. Antibodies used for each experiment are referenced in online supplemental table 2. Cells were acquired using the Gallios G flow cytometer and Kaluza Gallios software or the DxFlex flow cytometer and CytExpert software. For imaging flow cytometry, cells were acquired using the ImageStreamX Mark II and the IDEAS software. Compensation was performed using single stainings of respective cell types treated with 5% Tween 20 for 5 min for viability stains, as well as single stainings of UltraComp eBeads compensation beads with respective antibody stains. Gating, compensation matrix calculation and data visualization was performed in Kaluza Analysis software.

Microscopic images of living cultures were taken with the Axiovert 40 CFL microscope, and the AxioCam ICC 3. H&E and IF staining was imaged using the Vectra Polaris Automated Quantitative Pathology Imaging System.

### Statistical analysis

Differences between the two groups were analyzed by Student’s t-test. Differences between three or more groups were analyzed by two-way analysis of variance and subsequent Tukey’s multiple comparisons. Statistical computing as well as scatter plot visualization was performed in GraphPad Prism software.

## Results

### CAFs instruct monocytes to gain a TAM-like phenotype

**Figure 1:**
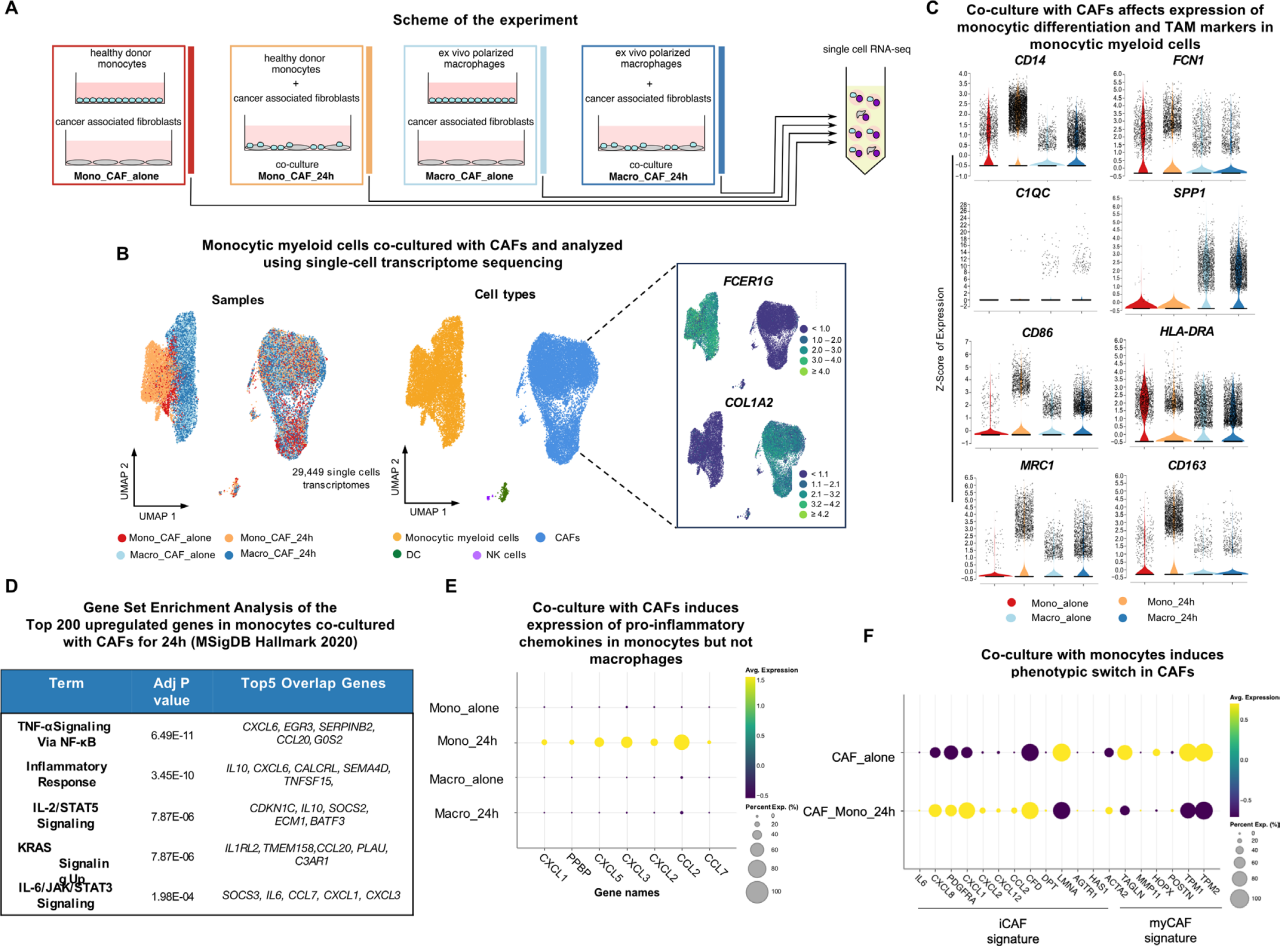
Single-cell transcriptomics of CAF co-cultures with monocytic myeloid cells reveal CAF-induced TAM-like phenotypes. (**A**) Schematic representation of the experimental set-up. Cultures were incubated in the basal medium. (**B**) Visualization of the data set (n=29,449 single cells) using Uniform Manifold Approximation and Projection based on the sample type and cell type features. Overlaid expression of the markers FCER1G and COL1A2 (inside the box). (**C**) Violin plots of monocytic differentiation and TAM markers expression. Data is represented based on the Z-score of expression, sorted by the sample type, each dot represents one cell. (**D**) Gene Set Enrichment Analysis using Enrichr (based on MSigDB 2020 Database) of the top 200 upregulated genes in monocytes co-cultured with CAFs for 24 hours. (**E**) Dot plots showing average expression of chosen pro-inflammatory chemokines upregulated in monocytes co-cultured with CAFs for 24 hours. (**F**) Dot plots showing average expression of iCAF and myCAF genes expressed in CAFs cultured alone and co-cultured with monocytes for 24 hours. CAF, cancer-associated fibroblast; DC, dendritic cell; iCAF, inflammatory cancer associated fibroblast; myCAF, myofibroblastic cancer associated fibroblast; NK, natural killer; TAM, tumor-associated macrophage.

Classification of TAM subtypes is a frequently revisited topic in the field of tumor biology. Current subclassifications drift away from a separation of TAMs expressing an M1 or M2 signature, based on findings that marker genes of both are frequently found on the same myeloid cell in the TME of several cancer types.^33^ More recent classifications of TAM subsets in CRC are designated based on the expression of markers including *FCN1*, *SPP1* and *C1QC*.^34 35^ However, studies to identify their developmental origin or assigning functional properties to these subsets in humans are limited. To test whether monocytes or macrophages are differentially influenced by a co-culture with primary CAFs, we isolated circulating monocytes from peripheral blood, as an *ex vivo* surrogate for tumor-naïve monocytic cells infiltrating the TME. In parallel, we examined the effects of CAF co-culture on mature macrophages, as a proxy for tissue-resident macrophages entering a tumor. Monocultures of CAFs, monocytes and macrophages, as well as co-cultures of either monocytes or macrophages with CAFs for 24 hours were subjected to scRNA-seq (figure 1A). Using UMAP analysis of cells pooled from all conditions we identified two primary clusters: *FCER1G*+ monocytic myeloid cells (including monocytes and macrophages) and *COL1A2*+ CAFs (figure 1B), along with minuscule clusters of *KLRC2*+ natural killer cells (cells) and *CCL19*+ dendritic cells (online supplemental figure S1A, online supplemental table 5). The presence of these small clusters is a likely consequence of impurities from the negative selection of CD14+ cells from the human blood sample. Our data revealed that CAFs significantly influenced TAM marker expression in monocytes but not in macrophages (figure 1C, online supplemental figure S1B). On co-culture, monocytes upregulated the expression of differentiation and activation markers, including *CD14*, *FCN1*, *CD86*, as well as markers associated with an immunosuppressive phenotype like *MRC1* (which encodes CD206) and *CD163*. In our data set, only differentiated macrophages expressed *SPP1* while *C1QC* was expressed by a small fraction of macrophages, regardless of CAF presence (figure 1C). To gain a deeper understanding of the phenotype induced by CAFs in activated monocytes, we selected the top 200 upregulated genes and conducted GSEA. The analysis revealed a highly significant enrichment for genes contributing to an inflammatory response in monocytes, including signals associated with TNF-α, IL-2/STAT5, KRAS, IL-6/JAK/STAT3, and inflammatory responses (figure 1D). In contrast, enrichments for genes contributing to lipid and lipoprotein metabolism were highly significant among the top 200 downregulated genes in monocytes (online supplemental figure S1C). Focusing on the secreted proteins of CAF-instructed monocytes, we detected a strong expression of chemokines, including *CXCL1*, *PPBP* (*CXCL7*), *CXCL5*, *CXCL3*, *CXCL2*, *CCL2*, and *CCL7* (figure 1E). Likewise, we observed an upregulation of genes in CAFs that gave rise to similar pathway enrichments as in monocytes (TNF-α, IL-6/JAK/STAT3) and the induction of the same chemokines (*CXCL6*, *CXCL1*, *CXCL3*, *CXCL2*, *CCL2*), suggestive of feedback loops to create chemo-attractive gradients towards myeloid cells (online supplemental figure S1D,E). The analysis of our data set in respect to inflammatory cancer-associated fibroblast (iCAF) and myofibroblastic cancer-associated fibroblast (myCAF) signatures^36^ revealed that CAFs exhibit plasticity, shifting from an myCAF to an iCAF phenotype in the presence of monocytes (figure 1F). This plasticity, influenced by monocyte-CAF interactions, may contribute to the induction of the inflammatory CAF phenotype in the tumor periphery, a phenomenon observed across various cancer types.^37^ More detailed analysis of the CAF subset in our scRNA-seq data revealed a high degree of CAF heterogeneity (online supplemental figure S2A–D), with monocyte interaction significantly altering the proportions of specific CAF clusters (online supplemental figure S2C,D), where clusters 2, 3 and 9 were proportionally more present in co-cultured monocytes and characterized by genes contributing to oxidative phosphorylation, epithelial to mesenchymal transition (EMT), TNF signals and other key protumorigenic signaling pathways. In contrast, clusters 4, 8 and 12 were mainly present in CAFs cultures without monocytes and were characterized by increased expression of genes associated with cell proliferation (online supplemental figure S2D–F). This suggests that monocyte co-culture leads to proliferation arrest and a switch to more protumorigenic and inflammatory transcriptional programs in the CAF population.

In summary, our findings indicate that primary CAF interactions with monocytes, but not differentiated macrophages, induce wide-spread phenotypic changes in both cell types, including the upregulation of markers associated with immature macrophages and numerous pro-inflammatory signals and cytokines known to be associated with TAMs *in vivo*.

### CAFs critically drive a functional TAM-like phenotype in CRC organoid triple cultures

**Figure 2:**
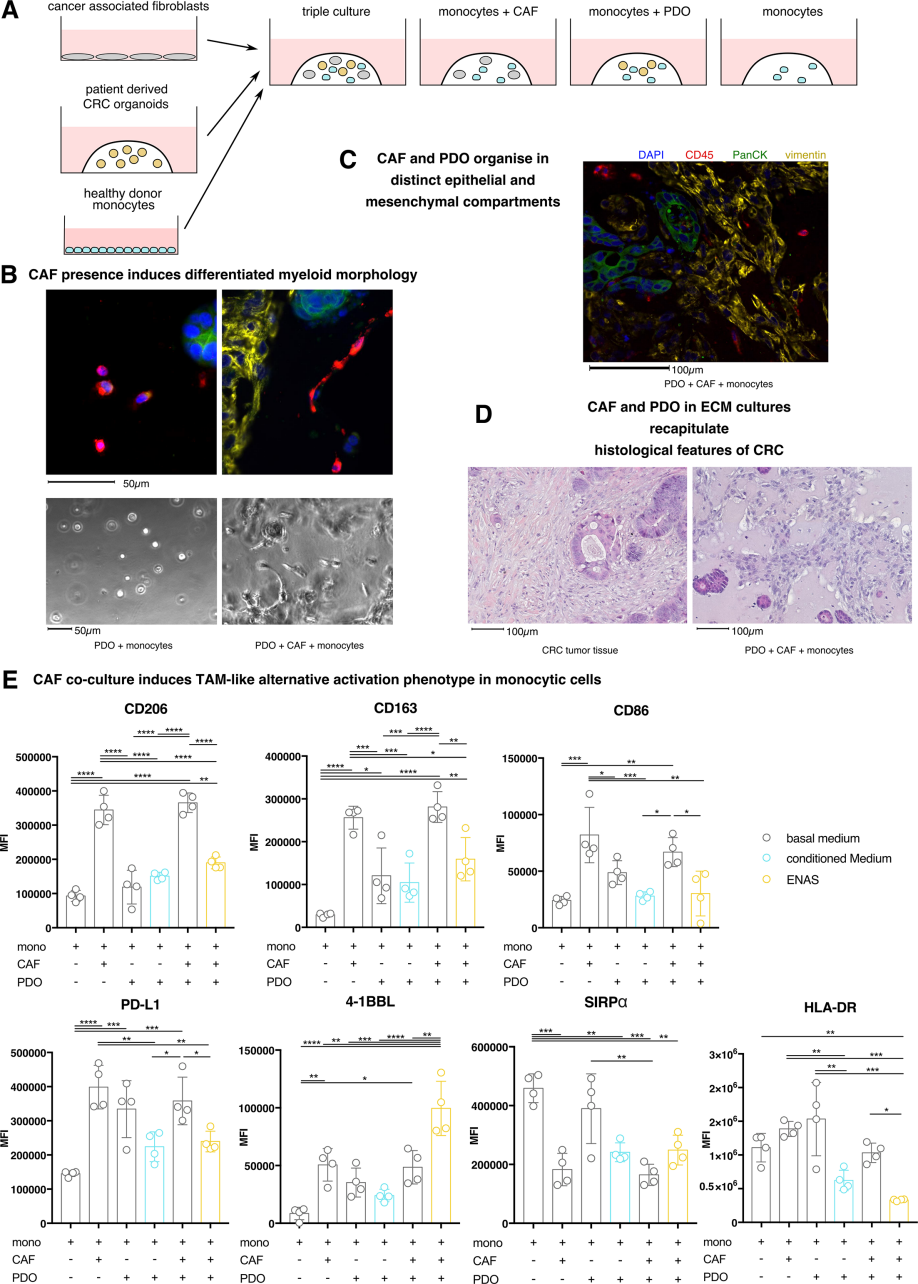
Monocyte differentiation to TAM-like monocytic myeloid cells in three-dimensional co-cultures with CRC organoids depends on the physical presence of CAFs. (**A**) Schematic representation of the experimental set-up. (**B**) Immunofluorescence (IF) staining of FFPE sections of a CAF, monocyte and CRC organoid triple culture 72 hours after seeding and culture in basal medium, as well as imaging unstained cultures. Upper images depict IF (DAPI, blue; CD45, red; PanCK, green). Lower images depict the brightfield of live cultures. Monocytes in focus, depicting their morphology in the respective culture conditions. Culture conditions and scale bar are indicated below. (**C**) Immunofluorescence staining of FFPE sections of a co-culture set-up of monocytes, PDOs and CAFs cultured for 72 hours in basal medium (DAPI, blue; vimentin, yellow; CD45, red; PanCK, green). (**D**) H&E staining of FFPE sections of a co-culture set-up of monocytes, PDOs and CAFs cultured for 72 hours in basal medium as well as a representative CRC tumor. (**E**) Flow cytometry quantification of CD206, CD163, CD86, HLA-DR, PD-L1, SIRPα and 4-1BBL median fluorescence intensity (MFI) on macrophages on indicated co-cultures in various media for 3 days. ns p>0.05; *p≤0.05; **p≤0.01; ***p≤0.001; ****p≤0.0001. CAF, cancer-associated fibroblast; CRC, colorectal cancer; DAPI, 4',6-diamidino-2-phenylindole, dihydrochloride; ECM, extracellular matrix; FFPE, formaldehyde-fixed paraphin embedded; PDO, patient-derived organoid; TAM, tumor-associated macrophage.

Our co-culture of CAFs with monocytes revealed a hitherto unknown influence of human CAFs on the phenotypic appearance of tumor naïve monocytes. However, to further extend the *in vitro* co-culture to more closely reflect the cellular complexity and relationships within the tumor ecosystem, we generated co-cultures of primary CRC organoids with CAFs and monocyte-derived TAM-like cells. As signals from CAFs contributed to the generation of TAM-like phenotypes, we first assessed the significance of CAF presence in 3D PDO-monocyte organotypic co-cultures. Primary cells were cultured in basal medium submerged ECM for 3 days, comprising CRC PDOs, monocytes and CAFs in various combinations (figure 2A). In the absence of CAFs, monocytes co-cultured with PDOs exhibited round morphology (figure 2B). The addition of CAFs induced a visible change in the morphology of the monocytes (figure 2B). To further characterize epithelial and mesenchymal compartments of the co-cultures, we used histology and IF staining and observed a high density of CAFs within the ECM scaffold resulting in the clustering of CAFs into a well-defined mesenchymal compartment, resembling characteristics seen in the patient tumors (figure 2C,D). Monocytic myeloid cells were found in the mesenchymal compartment, as well as in contact with the outer layer of epithelial tumor cells (figure 2C).

Next, we analyzed phenotypic markers of myeloid cells in organotypic co-cultures of PDOs and monocytes in the presence or absence of CAFs via flow cytometry. To study the influence of culture media on the TAM-like phenotype, cultures were grown (1) in the “gold-standard” ENAS medium, which is routinely used in PDO-immune co-cultures but due to complex additives (online supplemental table 6) may interfere with immune cell interactions, (2) in advanced DMEM/F12, described as basal medium, which should have little modulating effects on myeloid cells, or (3) in CAF-conditioned basal medium. Compared with the culture of monocytes alone, the addition of CAFs to the culture-induced upregulation of TAM phenotype markers CD206, CD163 and PD-L1 as well as co-stimulating molecules CD86 and 4-1BBL (figure 2E). SIRPα was downregulated by CAF presence and HLA-DR expression was found unchanged (figure 2E). Effects on all markers remained unchanged after the addition of PDOs, completing the triple culture together with CAFs and monocytes. The addition of PDOs to monocyte cultures alone enhanced PD-L1 expression, but had no significant effect on other markers (figure 2E). Importantly, culture of PDOs, CAFs and monocytes in ENAS medium had an overall suppressive effect on the upregulation of most phenotypic markers as compared with basal medium (figure 2E). Of note, only the expression of 4-1BBL was upregulated by ENAS (figure 2E). Additionally, basic markers of monocytic myeloid lineage and function, CD14 and CD64, were downregulated in triple cultures incubated in ENAS medium (online supplemental figure S3). Additional controls revealed, that in the absence of CAFs ENAS medium induced upregulation of CD86, HLA-DR and PD-L1, downregulation of 4-1BBL and no changes in CD206, CD163 and SIRPα as compared with monocyte culture in conditioned medium (online supplemental figure S4).

Interestingly, the addition of a CAF-conditioned medium to a co-culture of PDOs and monocytes was not able to stimulate marker gene expression (with the exception of HLA-DR and SIRPα, which were found downregulated). However, the stimulatory effect was less pronounced as compared to co-culture with CAFs. This is suggestive of a requirement for direct cell-to-cell contacts or dynamic cytokine communication with CAFs to induce this quantitative difference in marker gene expression on myeloid cells (figure 2E). In accordance with our scRNA-seq data, the addition of CAFs lead to an upregulation of CD14 as well as CD64 expression (online supplemental figure S3), demonstrating the role of CAFs in the maintenance of the highly plastic monocytic cell identity.

Taken together our results demonstrate that primary CAF-PDO-monocyte co-cultures recapitulate many parameters characteristic of TAMs. Our human, primary triple-culture identified CAFs to orchestrate instructive signals leading to incomplete macrophage differentiation. Moreover, CAFs induce morphological changes while upregulating monocyte markers. Thus, direct and indirect communication with CAFs maintains their plastic nature and confers their susceptibility to receiving signals that implement a TAM-like immunosuppressive program.

### Single-cell transcriptome sequencing reveals specific therapy-response phenotypes in the triple culture model

**Figure 3:**
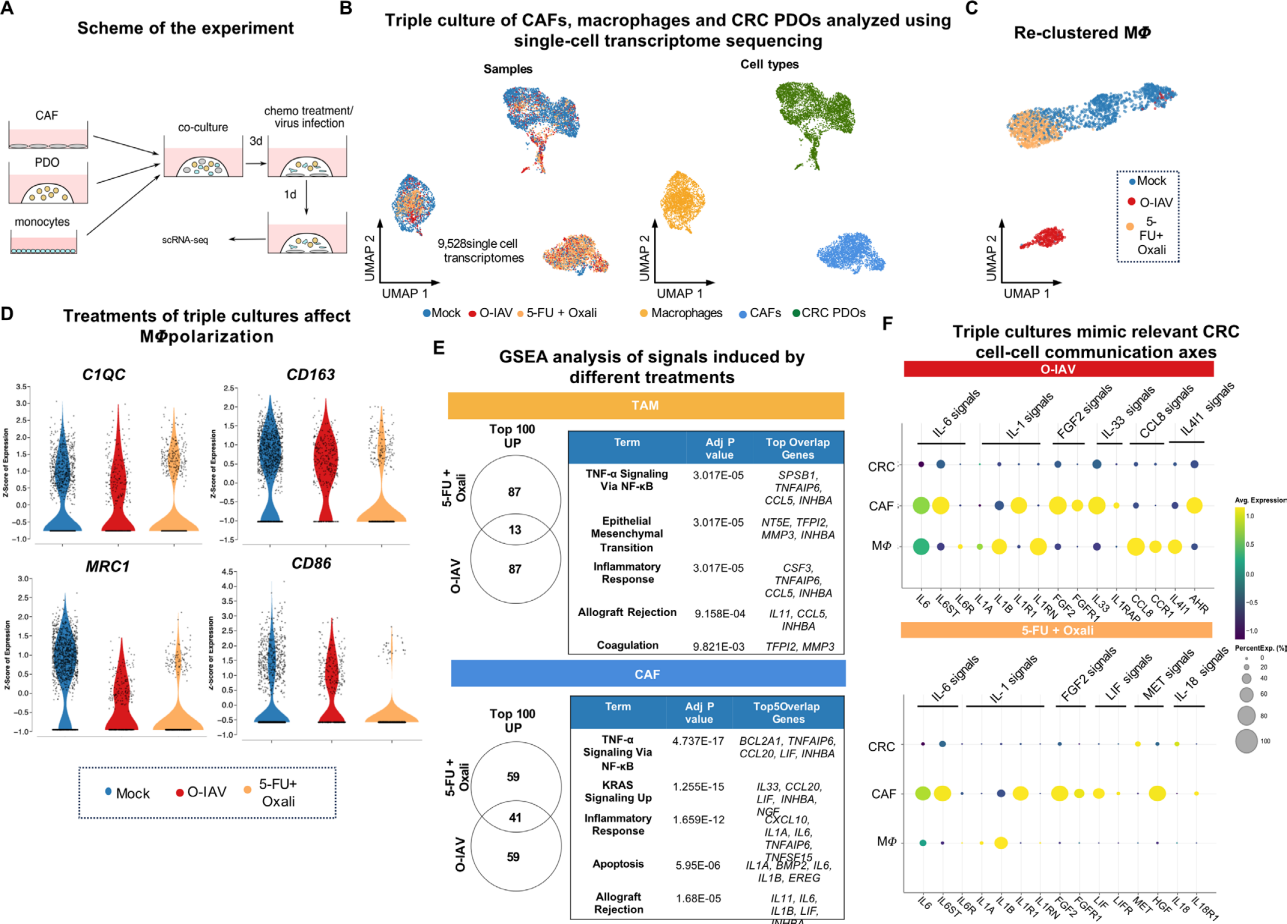
Single-cell transcriptomics of triple cultures enables the definition of distinct myeloid cell states and associated RNA expression profiles induced in response to treatment with chemotherapy or O-IAV. (**A**) Experimental set-up. Co-cultures were incubated in the basal medium. (**B**) Visualization of the data set (n=9,528 single cells) using UMAP projection based on the sample type and cell type features. (**C**) UMAP projection of re-clustered TAM. (**D**) Violin plots of monocytic differentiation and TAM marker expression. Data is represented based on the Z-score of expression, sorted by the sample type, each dot represents one cell. (**E**) Venn diagrams (visualized using Venny V.2.0) and Gene Set Enrichment Analysis (GSEA) using Enrichr (based on MsigDB 2020 database) of the common therapy response signatures based on a comparison of the top 100 (TAM) and top 100 (CAFs) upregulated genes. (**F**) Dot plots showing average expression of ligands and receptors upregulated by O-IAV and chemotherapy represented in all three cell types. CAF, cancer-associated fibroblast; CRC, colorectal cancer; O-IAV, oncolytic influenza A virus; PDO, patient-derived organoid; scRNA-seq, single-cell RNA sequencing; TAM, tumor-associated macrophage; UMAP, Uniform Manifold Approximation and Projection; 5-FU, 5-fluorouracil.

We next tested our set-up in response to oxaliplatin in combination with 5-FU, which are used in clinical practice as first-line chemotherapy for CRC. Chemotherapeutic dosage was chosen according to maximal plasma concentrations determined in pharmacokinetic studies of patient treatments.^38 39^ Due to the known strong immunogenic effects of viruses on monocytic myeloid cells, we used an O-IAV as an additional treatment for reference. First, we tested the capacity of this oncolytic virus to infect tumor organoids. Indeed, O-IAV was able to infect tumor organoids by passive diffusion into the ECM gel (online supplemental figure S5). We further tested its capacity to infect other cell types in a 3-day triple culture. We could observe infection of monocytic cells and PDOs, but not of CAFs within 48 hours post treatment (online supplemental figure S6). To examine the effects of chemotherapy and O-IAV on the phenotypes of the cells in our triple culture model, we performed single-cell transcriptomic analysis. The cultures were treated for 24 hours and then subjected to single-cell sequencing (figure 3A). The UMAP projection of 9,528 sequenced, good quality cells revealed that the clustering was driven by cell types rather than the treatment regimen (figure 3B). However, when we re-clustered only TAM-like cells they displayed a distinct phenotype according to treatment (figure 3C). Moreover, our data indicates that different treatments induced specific polarization patterns in TAM-like cells. Unlike the double cultures of CAFs and monocytes (figure 1C), the triple culture induced polarization of myeloid cells towards *C1QC*+ TAM like cells, indicating a possible role of the colorectal cell compartment in shaping this macrophage phenotype (figure 3D). While *SPP1*+ macrophages were largely absent in the triple culture setting (online supplemental figure S7A), we observed high expression of *MRC1* (CD206), *CD86*, and *CD163* markers in mock-treated cells, which decreased with treatments, particularly after treatment with chemotherapy (figure 3D). Following treatment with O-IAV, we observed a strong upregulation of canonical type I and II interferon (IFN) signaling, leading to the upregulation of genes including *ISG15*, *MX1*, *IFIT1*, and *OAS1* in all three cell types (online supplemental figure S7B). When comparing the strong inflammatory phenotype induced by oncolytic virotherapy with the phenotype induced by chemotherapy in TAM-like cells, we identified common signals related to “Epithelial Mesenchymal Transition”, “Inflammatory Response”, and “TNF-α Signaling via NF-κB” with high significance. This finding suggests that chemotherapy-induced phenotypes in TAM-like cells have an immunomodulatory aspect in our triple-culture setting (figure 3E). Cluster analysis uncovered high plasticity and strong polarization of the TAM-like cells, manifested by a reduction in myeloid cell heterogeneity under both treatments. Oncolytic virotherapy leads to a proportional increase of clusters 4 and 7, resembling inflammatory clusters with a prominent IFN and TNF-α signature (online supplemental figure S8A–D). In contrast, chemotherapy treatment of TAM-like cells resulted in a proportional increase of clusters 0 and 1, characterized by the expression of specific genes downstream of TNF-α signaling and genes involved in the epithelial-to-mesenchymal transition and cell cycle (online supplemental figure S8). Notably, the quality of the response to O-IAV and chemotherapy was more similar in CAFs as compared with TAM-like cells, with a strong induction of TNF signaling (figure 3E, online supplemental figures S7C and S9), putting further emphasis on the specificity and importance of the CAF compartment in the therapy response.

As the induction of the immunosuppressive program in monocytes by CAFs required a combination of direct cell-to-cell contact and secreted factors, we next focused on the regulation of genes encoding secreted factors. The inclusion of three cell types in the culture system allowed us to recapitulate essential cell-to-cell communication axes within the CRC TME. We observed the induction of inflammatory signals in the CRC PDOs, including upregulation of *IL-6*, *CXCL6*, *IL-1B*, and *TNFAIP6* expression, in response to both treatments (online supplemental figure S7D). Additionally, both treatments induced an upregulation of *IL-6* by CAFs and TAM-like cells, along with the expression of the *IL-6* receptors, *IL-6R* and *IL-6ST* (Gp130), in a subset of cells in each cell type (figure 3F). Another strongly upregulated signal by both treatments was *IL-1* signaling. *IL-1A* and *IL-1B* were found to be expressed by CAFs and TAM-like cells, with CAFs being recipients of the signal, as reflected by their expression of *IL-1R1* (figure 3F). Interestingly, O-IAV treatment additionally induced strong expression of *IL-1RN* in TAM-like cells, encoding the IL-1 receptor antagonist protein, suggestive of an IL-1-inhibitory feedback loop to fine-tune the response (figure 3F). Additionally, we identified another communication axis in the triple culture: the *FGF2-FGFR2* interaction, with CAFs expressing both *FGF2* and *FGFR2*, being both senders and receivers of this autocrine stimulation, previously implicated in CRC therapy resistance.^40 41^ Finally, we observed several treatment-specific effects on the level of cell–cell communication, including clinically relevant signals coming from *IL-33-IL-1RAP*, *CCL8-CCR1*, and *IL-4l1-AHR* interactions induced by the oncolytic virus treatment, and *LIF-LIFR*, *MET-HGF*, *IL-18-IL-18R1* triggered by chemotherapy (figure 3F).^42^,^47^

In summary, our data demonstrate that the complex primary CRC organoid triple cultures model multiple molecular aspects of treatment responses. We provide evidence of an immunogenic signal generated by chemotherapy, which, at least in part, counteracted the CAF-induced, immunosuppressive program in TAM-like cells. This is in line with the observed onset of inflammatory signaling circuits.

### Chemotherapy treatment counteracts the CAF-induced immunosuppressive program in TAM-like cells

**Figure 4:**
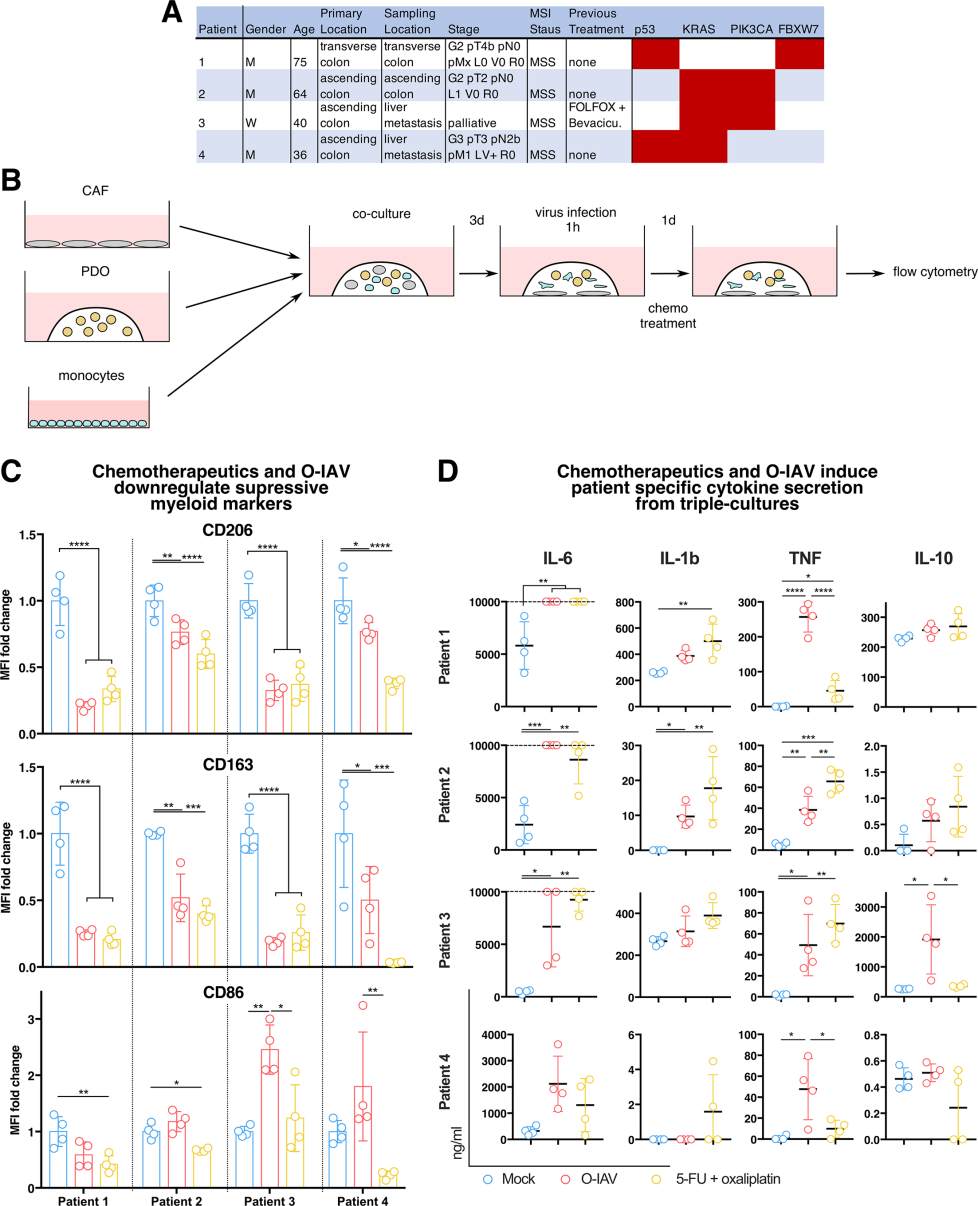
Chemotherapeutics and oncolytic influenza A viruses induce repolarization of tumor-associated macrophage-like cells in primary triple culture assays. (**A**) Table of patient characteristics. (**B**) Experimental set-up. Cultures incubated in basal medium. (**C**) Quantification of CD206, CD163 and CD86 median fluorescence intensity (MFI) normalized to the corresponding patient’s MFI from the untreated condition, measured on macrophages from treated triple cultures. (**D**) Quantification of cytokine concentrations (ng/mL) in the supernatants collected from C, determined via LEGENDplex assay. Upper detection limits are indicated by a dashed line. ns p>0.05; *p≤0.05; **p≤0.01; ***p≤0.001; ****p≤0.0001. All color-coded scatter-plot circles represent measurements from individual donors. CAF, cancer-associated fibroblast; IL, interleukin; O-IAV, oncolytic influenza A virus; PDO, patient-derived organoid; 5-FU, 5-fluorouracil; TNF, tumour necrosis factor.

Our single-cell sequencing data revealed treatment-dependent effects on TAM-like cells and CAFs in our triple culture model. We next sought to validate the decreased expression of the immunosuppressive markers CD206 (*MRC1*), CD163 and CD86 in TAM-like cells on the protein level. We treated cultures from four patients with distinct CRC (figure 4A) with the indicated therapeutics for 24 hours and assessed the expression of immunosuppressive markers in TAM-like cells using flow cytometry (figure 4B). In line with our observations on mRNA level, both chemotherapy and virotherapy downregulated the immunosuppressive markers CD206 and CD163 (figure 4C). However, we observed a trend of the O-IAV to upregulate the expression of the T-cell-activating ligand CD86, whereas chemotherapy suppressed CD86 expression (figure 4C).

In order to validate that the observed effects remain relevant in myeloid cells from the peripheral blood of patients with CRC with a current tumor burden, the above assays were performed with monocytes extracted from the peripheral blood of patients with CRC (online supplemental figure S10). We were able to observe similar deregulation of the TAM markers CD206, CD163 and CD86 as observed with healthy donor monocytes (online supplemental figure S10). Furthermore, we observed higher susceptibility to treatment-induced cell death in TAM-like cells derived from patients with CRC compared with healthy donors (online supplemental figure S11).

Our single-cell transcriptomics data showed a treatment-dependent upregulation of target genes downstream of the IL-6 and IL-1β signaling cascade in both CAFs and TAM-like cells (figure 3). Besides these pro-inflammatory signals, we detected gene activity downstream of the TNF pathway as well as upregulation of STAT3-dependent genes, which could be attributed to signaling downstream of IL-6 and/or IL-10. Following this notion, we probed the supernatants collected from the treated co-cultures to test levels of these cytokines and additional clinically relevant cytokines.^48^ Both treatments induced the secretion of pro-inflammatory cytokines IL-1β, IL-6 and TNF in patients with primary tumor 1 and 2 (figure 4D). IL-6 and TNF were also induced by both treatments in patient 3, while only the O-IAV induced TNF secretion in patient 4 (figure 4D). The anti-inflammatory cytokine IL-10 was only significantly induced by oncolytic virotherapy in patient 3 (figure 4D).

Taken together, we observed significant downregulation of immunoinhibitory markers by both treatments. Moreover, we observed a patient-dependent increase in the secretion of inflammatory cytokines, demonstrating that our triple culture model faithfully recapitulates the complexity of cell intrinsic and extrinsic immunomodulatory regulators in response to treatments *in vitro*.

### Chemotherapeutic and O-IAV treatments of PDOs induce patient-dependent susceptibility to phagocytosis by TAMs

Phagocytosis is a key feature of monocyte and macrophage function. We hypothesized that treatment of cancer cells with oxaliplatin, 5-FU or a combination of them would induce changes in cancer cell susceptibility to be phagocytosed by monocytes. In line with our previous finding that CAF-instructed monocytes undergo phenotypic changes, we further examined whether CAFs affect the phagocytic abilities of monocytes in our triple culture model.

**Figure 5:**
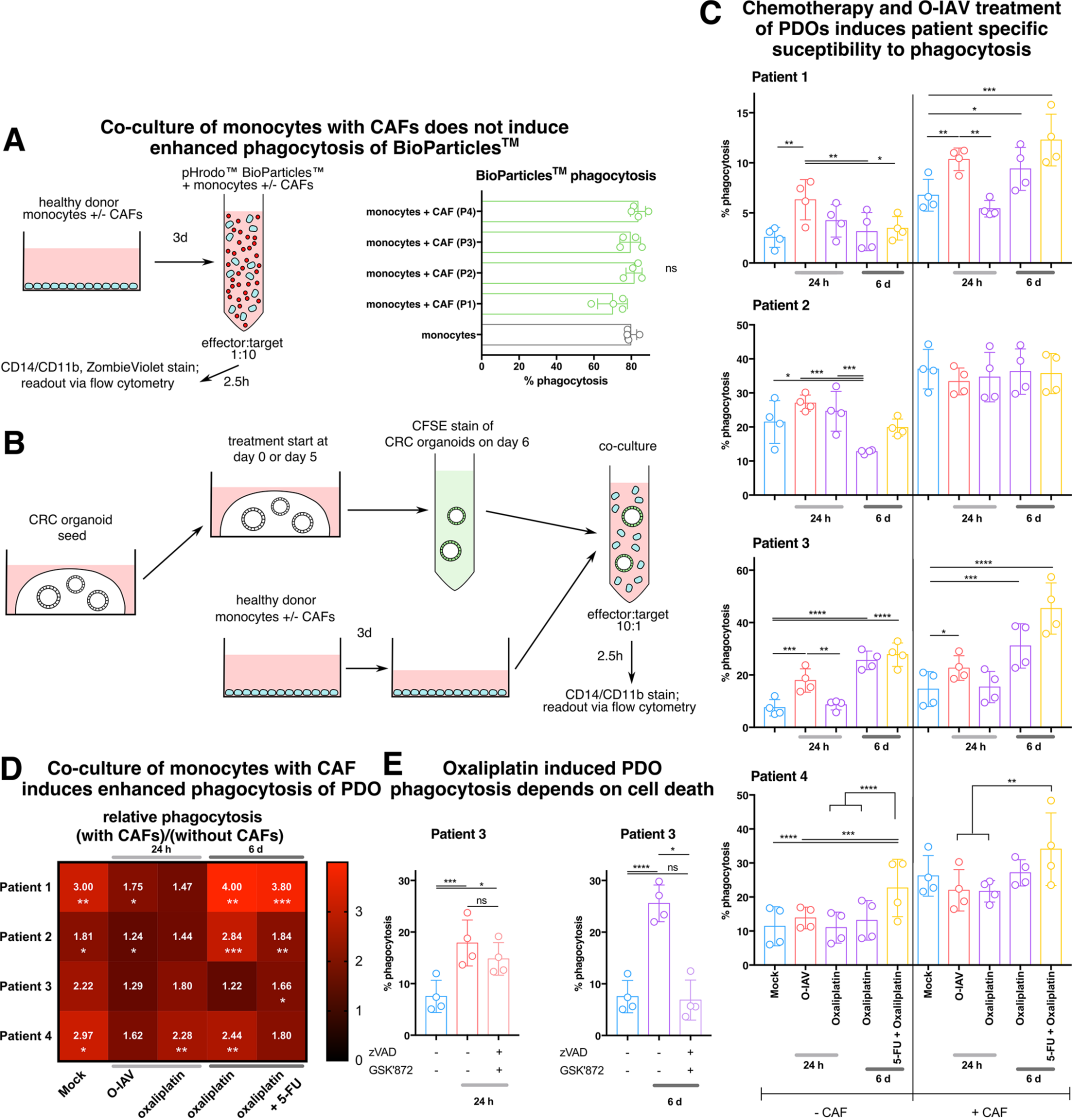
Oncolytic viruses and chemotherapy induce varying, patient-dependent levels of CRC organoid phagocytosis via distinct mechanisms. (**A**) Flow cytometry analysis of phagocytosis assay using BioParticles. Monocytes were incubated alone or in the presence of patient with CRC-derived CAFs isolated from four patients for 3 days and subsequently co-cultured with BioParticles for 2.5 hours. (**B**) Scheme of experimental set-up of phagocytosis assay after treatments. Organoids were seeded simultaneously and subjected to medium changes introducing the indicated therapeutics or viral infections, leading to treatment durations of either 6 days or 1 day before CFSE stain and 2.5 hours culture with monocytes or a CAF/monocyte mixture. (**C**) Flow cytometry measurements of phagocytotic events detected as CD14/CD11b^+^CFSE^+^ cells. (**D**) Heat-map showing the change in phagocytosis after CAF introduction for each condition depicted in C. (**E**) Flow cytometry analysis of phagocytosis under viral treatment and oxaliplatin treatment in the presence or absence of inhibitors of cell death (10 µM zVAD, 2 µM GSK’872). ns p>0.05; *p≤0.05; **p≤0.01; ***p≤0.001; ****p≤0.0001. All color-coded scatter-plot circles represent measurements from individual donors. CAF, cancer-associated fibroblast; CFSE, carboxyfluorescein diacetate succinimidyl ester; CRC, colorectal cancer; O-IAV, oncolytic influenza A virus; PDO, patient-derived tumor organoid; 5-FU, 5-fluorouracil.

First, we ruled out that co-culture with CAFs of different patients resulted in different phagocytic activity of the TAM-like cells using beads (figure 5A). Before analyzing the phagocytic capabilities of the TAM-like cells, we determined CRC organoid viability under all treatments over a time course of 1–6 days (online supplemental figure S12). O-IAV treatment induced cell death within 24 hours in two patients (online supplemental figure S12). While we observed some patient heterogeneity in the treatment responses, all PDOs responded to a 6-day treatment with a combination of 5-FU and oxaliplatin (online supplemental figure S12).

We next established an assay set-up to detect phagocytic events of CRC PDOs by TAM-like cells (figure 5B). Phagocytotic engulfment of cancer cells was confirmed for a representative patient via imaging-flow-cytometry (online supplemental figure S13). Oxaliplatin alone did not induce phagocytosis of any PDO culture after 1 day of treatment (figure 5C). In contrast, O-IAV treatment induced phagocytosis in two out of four patients (patients 1 and 3). Only the PDOs of patient 3 displayed increased cell death at this time point (figure 5C, online supplemental figure S12). Chemotherapy-treated PDOs induced significant phagocytosis by CAF-educated TAM-like cells after 6 days of culture in three patients, with 5-FU being necessary for phagocytosis induction in patient 4 and oxaliplatin alone being sufficient in patients 1 and 3 (figure 5C). Overall, there was significant patient-driven heterogeneity of phagocytosis induction between different PDOs. Interestingly, in patients 3 and 4 every significant increase in phagocytosis coincided with an increase in cell death at the same time point, whereas in patients 1 and 2 phagocytosis occurred independently from cell death induction by the respective treatments at the corresponding time points (figure 5C, online supplemental figure S12). We next directly compared phagocytosis measured for each treatment and patient depending on CAF presence, by calculating the ratio of phagocytosis measured with CAF co-cultured monocytes compared with freshly isolated ones (figure 5D). CAF co-culture of myeloid cells led to significantly increased phagocytosis in 60% of all conditions, but an upward trend was observed in all comparisons (figure 5D). This effect was more pronounced in 6-day chemotherapy-treated conditions.

As the phagocytosis observed in this assay may to a certain extent comprise efferocytosis of dead cellular material,^49^ we evaluated the mechanism underlying phagocytosis of PDOs treated with chemotherapy and O-IAV. To this end, we performed the treatment of PDOs in the phagocytosis assay in the presence versus absence of inhibitors of cell death pathways. We observed that cell death inhibition abrogated monocyte phagocytosis of 6-day oxaliplatin-treated PDOs but at the same time it had no significant effect on phagocytosis of O-IAV infected PDOs, 24 hours after infection (figure 5E). This indicates, that phagocytosis of PDOs after 24 hours, as seen in viral treatment, comprises a true effector mechanism of monocytic myeloid cells towards living cancer cells, whereas phagocytic events observed on a 6-day treatment reflected the phagocytes’ natural purpose of dead cell clearance.

Taken together, using our *in vitro* model system, we identified chemotherapeutic treatment with 5-FU and oxaliplatin to induce phagocytosis in TAM-like cells within 24 hours of co-culture. In contrast, cancer cell lysis in the wake of infection with the oncolytic virus did not result in an increase in phagocytosis, suggesting that the effect of the two treatments on cancer cells is substantially different in regards to inducing phagocytosis by TAM-like cells.

## Discussion

We recently demonstrated that CAFs provide a sufficient physiological condition to support CRC PDO differentiation without similar supplemented media.^18^ Our results now indicate that CAF presence not only supports differentiation and health of PDOs but also supports cancer-associated re-programming of monocytic myeloid cells, and therefore eliminates the need for artificial growth factor and compound supplementation for maintenance of these cell types within the organotypic culture. This finding enables more pathophysiological culture systems and therefore has major implications for the design of future ex vivo cancer organotypic culture experiments in drug testing and precision medicine.

To the best of our knowledge, there is no data in clinically relevant human primary culture systems examining immunomodulatory effects in the immediate 24-hour aftermath of chemotherapy initiation in CRC. The results obtained in this study point towards the reversal of inhibitory myeloid cell phenotypes shortly after the onset of chemotherapeutic treatment in CRC, contrasting with known myelosuppressive effects of long-term chemotherapy.^50^

Recent studies establishing the feasibility of co-culture systems of primary human PDOs and leukocytes in CRC have focused on T cells^14 15^ and their interaction with macrophages.^51^ Other recent studies addressing the cancer-TME relationship and communication have been conducted using murine cancer models and cells including macrophages and fibroblasts.^52^,^54^ In the current study, we demonstrate that culture media supplemented for organoid growth induces major phenotypic changes and possibly maturation deficits in monocytic myeloid cells in a primary human setting. Co-culture assays containing CRC-derived PDOs so far required media containing organoid growth factors and supporting compounds, which is necessary for organoid propagation and survival.^53^ To the mostly T-cell-focused studies conducted in the field of CRC biology so far, our study also adds the importance of cell-to-cell communication between CAFs and monocytic myeloid cells to orchestrate the signals leading to the immunosuppressive phenotypes observed in TAMs.^55 56^

TAMs are known as a heterogeneous population potentially comprised of tissue-resident macrophages and monocyte-derived macrophages. Recent studies showed that CAFs could educate monocytes towards immunoinhibitory phenotypes in breast cancer.^57^,^59^ Along these lines, we observed inhibitory phenotypes in our TAM-like cells, which developed after only 1 day of contact between monocytes and CAFs. In squamous cell carcinoma, a similar instruction of monocytes to TAM-like myeloid cells was observed and induced through IL-6 and granulocyte and macrophage colony-stimulating factor (GM-CSF) secretion by CAFs.^58^ CAF-educated monocytes exerted pro-tumorigenic and anti-inflammatory modulating effects on the immune microenvironment.^57 59 60^ Overall, we observed similar patterns of monocyte instruction by CAFs in CRC. However, the phenotypic plasticity observed for monocytes was absent after CAF co-culture with macrophages differentiated *in vitro* from the same monocyte precursor pool. Thus, macrophages appear to be significantly less responsive to CAF-derived immunosuppressive signals compared with circulating monocytes entering the tumor.

Immunogenic effects induced by chemotherapy treatment, at least in part, disturbs this immunosuppressive communication and renders the TAM-like cells in our culture model more active (ie, downregulation of CD206 and CD163) and more functional in regards to phagocytosis of chemotherapy-treated cancer cells. The immunogenic effects of chemotherapy on TAM-like cells were overall comparable to the treatment with an oncolytic virus, a potent immunostimulatory agent. However, in contrast to cell death induced by the oncolytic virus, the CAF-instructed TAM-like cells were more effective in phagocytosing cancer cells when cell death was a consequence of chemotherapy treatment. Phagocytosis of cancer cell material, dead or alive, is a key step in antigen presentation and therefore necessary for the adaptive immune response and T-cell-mediated tumor control.^61^ Moreover, phagocytosis of intact cancer cells in itself comprises an antitumor effector mechanism.^62^ Thus, our model successfully recapitulated phagocytosis in a primary CRC *ex vivo* co-culture. This is of high relevance for the development of drugs targeting phagocytosis in cancer, a topic that recently gained momentum,^62 63^ in need of complex systems like the one presented in this study, accurately recapitulating a functional phagocytic readout of primary cancer cells.

Our signaling network analysis discovered pathways induced by both chemotherapy and the O-IAV including IL-1, IL-6 and FGF2 signals. Previous studies on the CRC TME described IL-1 signaling as important in TAM-CAF communication, inducing immunosuppressive TAM features and protumorigenic effects in CRC.^64^ IL-6 was identified as a key signal in CRC development^65^ and CAF-derived IL-6 was shown to mediate monocyte differentiation towards TAMs^58^ as well as promoting CRC metastasis.^66^ Although IL-6/IL-6R-inhibiting compounds are in clinical development for various cancers,^67^ there is evidence that vaccination-based immunotherapy depends on IL-6 signaling in TAMs for optimal treatment efficacy.^68^ Our data is in line with the latter report and showed that IL-6 upregulation may be an enhancing factor of the applied treatments. Chemotherapy-specific pathways identified across cells of the triple culture included leukemia inhibitory factor (LIF), which was previously shown to push TAMs into building cytotoxic, T-cell-excluding microenvironments in glioblastoma,^69^ as well as EMT which is associated with unfavorable prognosis in CRC^70^ and IL-18, known to contribute to CRC regression.^71^ Furthermore, we identified TNF-mediated inflammatory responses as the most prominent immunogenic signaling pathway shared between O-IAV and chemotherapy in both TAM-like cells and CAFs. TNF appears to be a supportive factor in oncolytic viral treatment,^72 73^ but has been shown to negatively impact chemotherapy outcome in patients with CRC and animal models.^74 75^ Overall, there seems to be complex reciprocal cross-talk between the three cell types included in this culture, especially TAM-like cells and CAFs. This notion is further strengthened by our finding of reciprocal cluster shifts in the CAF population in response to monocyte co-culture (online supplemental figure S2). Single-cell transcriptomic approaches of CRC tumor samples have been used to inform possible myeloid-targeted treatments.^34^ Our triple culture model enables a more dynamic and functional discovery of myeloid-targeted therapies. Probing multiple patients under various treatments, using our approach, could lead to the discovery of potential compensatory/resistance mechanisms mediated by the TME. However, one limitation of the current study is a bias towards patients with later-stage CRC. Follow-up studies will need to address a larger patient heterogeneity.

Taken together, we here describe a novel PDO organotypic assay containing CAFs and TAM-like cells. The experiments shown create a baseline for both phenotypic and functional readouts in the monocytic myeloid compartment of CRC in primary cultures of patient cohorts. This proposed system enables the dissection of treatment response pathways in primary cultures and provides a tool for potential discoveries of drug combinations including myeloid immune checkpoint inhibitors^76^ and compounds inducing immunogenic cell death.^61^ Furthermore, its ability to preserve patient heterogeneity, as shown by our obtained cytokine profiles in response to treatments, provides the opportunity to model novel drug combinations in patient cohorts, as well as drug screens for individual patients in precision medicine approaches by probing immunogenic effects on myeloid cells.https://www.dropbox.com/scl/fi/qlnxquhj87wag7w2imtn5/Supplementary_Tables.zip?rlkey=c94dxwaof31uo56gsrkvmmevr&st=vy0kf7z6&dl=0.

## supplementary material

10.1136/jitc-2024-009494 online supplemental figure 1

10.1136/jitc-2024-009494 online supplemental table 1

## Data Availability

Data are available upon reasonable request.

## References

[1] Schouppe E, De Baetselier P, Van Ginderachter JA (2012). Instruction of myeloid cells by the tumor microenvironment: Open questions on the dynamics and plasticity of different tumor-associated myeloid cell populations. Oncoimmunology.

[2] Bonavita E, Galdiero MR, Jaillon S (2015). Phagocytes as Corrupted Policemen in Cancer-Related Inflammation. Adv Cancer Res.

[3] Yahaya MAF, Lila MAM, Ismail S (2019). Tumour-Associated Macrophages (TAMs) in Colon Cancer and How to Reeducate Them. J Immunol Res.

[4] Wang H, Tian T, Zhang J (2021). Tumor-Associated Macrophages (TAMs) in Colorectal Cancer (CRC): From Mechanism to Therapy and Prognosis. Int J Mol Sci.

[5] Hourani T, Holden JA, Li W (2021). Tumor Associated Macrophages: Origin, Recruitment, Phenotypic Diversity, and Targeting. Front Oncol.

[6] van de Wetering M, Francies HE, Francis JM (2015). Prospective derivation of a living organoid biobank of colorectal cancer patients. Cell.

[7] Bao Y, Wang G, Li H (2024). Approaches for studying human macrophages. Trends Immunol.

[8] Weeber F, van de Wetering M, Hoogstraat M (2015). Preserved genetic diversity in organoids cultured from biopsies of human colorectal cancer metastases. *Proc Natl Acad Sci U S A*.

[9] Tran L, Tezerji RS, Malla CUP (2022). Abstract A011: Epigenetic vulnerabilities in patient-derived colorectal cancer organoids. Cancer Res.

[10] Ooft SN, Weeber F, Dijkstra KK (2019). Patient-derived organoids can predict response to chemotherapy in metastatic colorectal cancer patients. *Sci Transl Med*.

[11] Yao Y, Xu X, Yang L (2020). Patient-Derived Organoids Predict Chemoradiation Responses of Locally Advanced Rectal Cancer. Cell Stem Cell.

[12] Ganesh K, Wu C, O’Rourke KP (2019). A rectal cancer organoid platform to study individual responses to chemoradiation. *Nat Med*.

[13] Mo S, Tang P, Luo W (2022). Patient‐Derived Organoids from Colorectal Cancer with Paired Liver Metastasis Reveal Tumor Heterogeneity and Predict Response to Chemotherapy. Adv Sci (Weinh).

[14] Dijkstra KK, Cattaneo CM, Weeber F (2018). Generation of Tumor-Reactive T Cells by Co-culture of Peripheral Blood Lymphocytes and Tumor Organoids. Cell.

[15] Chalabi M, Fanchi LF, Dijkstra KK (2020). Neoadjuvant immunotherapy leads to pathological responses in MMR-proficient and MMR-deficient early-stage colon cancers. Nat Med.

[16] Stadler M, Pudelko K, Biermeier A (2021). Stromal fibroblasts shape the myeloid phenotype in normal colon and colorectal cancer and induce CD163 and CCL2 expression in macrophages. Cancer Lett.

[17] Zhang R, Qi F, Zhao F (2019). Cancer-associated fibroblasts enhance tumor-associated macrophages enrichment and suppress NK cells function in colorectal cancer. Cell Death Dis.

[18] Atanasova VS, de Jesus Cardona C, Hejret V (2023). Mimicking Tumor Cell Heterogeneity of Colorectal Cancer in a Patient-derived Organoid-Fibroblast Model. Cell Mol Gastroenterol Hepatol.

[19] Kabiljo J, Laengle J, Bergmann M (2020). From threat to cure: understanding of virus-induced cell death leads to highly immunogenic oncolytic influenza viruses. Cell Death Discov.

[20] Kuznetsova I, Shurygina A-P, Wolf B (2014). Adaptive mutation in nuclear export protein allows stable transgene expression in a chimaeric influenza A virus vector. *J Gen Virol*.

[21] Ghiringhelli F, Bruchard M, Apetoh L (2013). Immune effects of 5-fluorouracil: Ambivalence matters. *Oncoimmunology*.

[22] Tian J, Zhang D, Kurbatov V (2021). 5-Fluorouracil efficacy requires anti-tumor immunity triggered by cancer-cell-intrinsic STING. *EMBO J*.

[23] Wu Y, Deng Z, Wang H (2016). Repeated cycles of 5-fluorouracil chemotherapy impaired anti-tumor functions of cytotoxic T cells in a CT26 tumor-bearing mouse model. BMC Immunol.

[24] Sansyzbay AR, Erofeeva MK, Khairullin BM (2013). An inactivated, adjuvanted whole virion clade 2.2 H5N1 (A/Chicken/Astana/6/05) influenza vaccine is safe and immunogenic in a single dose in humans. Clin Vaccine Immunol.

[25] Corrales L, Hipp S, Martin K (2022). LY6G6D is a selectively expressed colorectal cancer antigen that can be used for targeting a therapeutic T-cell response by a T-cell engager. *Front Immunol*.

[26] Venables WN, Ripley BD (2003). Modern Applied Statistics With S. Technometrics.

[27] Germain P-L, Lun A, Garcia Meixide C (2021). Doublet identification in single-cell sequencing data using *scDblFinder*. *F1000Res*.

[28] Korsunsky I, Millard N, Fan J (2019). Fast, sensitive and accurate integration of single-cell data with Harmony. *Nat Methods*.

[29] Korsunsky I, Nathan A, Millard N (2019). Presto scales wilcoxon and auroc analyses to millions of observations. Bioinformatics.

[30] Chen EY, Tan CM, Kou Y (2013). Enrichr: interactive and collaborative HTML5 gene list enrichment analysis tool. BMC Bioinformatics.

[31] Kuleshov MV, Jones MR, Rouillard AD (2016). Enrichr: a comprehensive gene set enrichment analysis web server 2016 update. *Nucleic Acids Res*.

[32] Xie Z, Bailey A, Kuleshov MV (2021). Gene Set Knowledge Discovery with Enrichr. *Curr Protoc*.

[33] Katkar G, Ghosh P (2023). Macrophage states: there’s a method in the madness. Trends Immunol.

[34] Zhang L, Li Z, Skrzypczynska KM (2020). Single-Cell Analyses Inform Mechanisms of Myeloid-Targeted Therapies in Colon Cancer. Cell.

[35] Cheng S, Li Z, Gao R (2021). A pan-cancer single-cell transcriptional atlas of tumor infiltrating myeloid cells. Cell.

[36] Yang D, Liu J, Qian H (2023). Cancer-associated fibroblasts: from basic science to anticancer therapy. Exp Mol Med.

[37] Öhlund D, Handly-Santana A, Biffi G (2017). Distinct populations of inflammatory fibroblasts and myofibroblasts in pancreatic cancer. J Exp Med.

[38] Casale F, Canaparo R, Serpe L (2004). Plasma concentrations of 5-fluorouracil and its metabolites in colon cancer patients. Pharmacol Res.

[39] Marty M (1989). L-OHP Phase I study. Debiopharm/Sanofi Rep No TDU3099.

[40] Szymczyk J, Sluzalska KD, Materla I (2021). FGF/FGFR-Dependent Molecular Mechanisms Underlying Anti-Cancer Drug Resistance. *Cancers (Basel*).

[41] Li P, Huang T, Zou Q (2019). FGFR2 Promotes Expression of PD-L1 in Colorectal Cancer via the JAK/STAT3 Signaling Pathway. J Immunol.

[42] Yeoh WJ, Vu VP, Krebs P (2022). IL-33 biology in cancer: An update and future perspectives. Cytokine.

[43] Kobayashi H, Gieniec KA, Lannagan TRM (2022). The Origin and Contribution of Cancer-Associated Fibroblasts in Colorectal Carcinogenesis. Gastroenterology.

[44] Carbonnelle-Puscian A, Copie-Bergman C, Baia M (2009). The novel immunosuppressive enzyme IL4I1 is expressed by neoplastic cells of several B-cell lymphomas and by tumor-associated macrophages. *Leukemia*.

[45] Liu J, Yu H, Hu W (2015). LIF is a new p53 negative regulator. *J Nat Sci*.

[46] Joosten SPJ, Spaargaren M, Clevers H (2020). Hepatocyte growth factor/MET and CD44 in colorectal cancer: partners in tumorigenesis and therapy resistance. Biochim Biophys Acta Rev Cancer.

[47] Li Y-P, Du X-R, Zhang R (2021). Interleukin-18 promotes the antitumor ability of natural killer cells in colorectal cancer via the miR-574-3p/TGF-β1 axis. Bioengineered.

[48] Bhat AA, Nisar S, Singh M (2022). Cytokine- and chemokine-induced inflammatory colorectal tumor microenvironment: Emerging avenue for targeted therapy. Cancer Commun (Lond).

[49] Boada-Romero E, Martinez J, Heckmann BL (2020). The clearance of dead cells by efferocytosis. Nat Rev Mol Cell Biol.

[50] Groeningen CJ v., Peters GJ, Leyva A (1989). Reversal of 5-Fluorouracil-Induced Myelosuppression by Prolonged Administration of High-Dose Uridine. JNCI J Natl Cancer Inst.

[51] Fang H, Huang Y, Luo Y (2022). SIRT1 induces the accumulation of TAMs at colorectal cancer tumor sites via the CXCR4/CXCL12 axis. Cell Immunol.

[52] Qin X, Sufi J, Vlckova P (2020). Cell-type-specific signaling networks in heterocellular organoids. Nat Methods.

[53] Below CR, Kelly J, Brown A (2022). A microenvironment-inspired synthetic three-dimensional model for pancreatic ductal adenocarcinoma organoids. *Nat Mater*.

[54] Qin X, Cardoso Rodriguez F, Sufi J (2023). An oncogenic phenoscape of colonic stem cell polarization. Cell.

[55] Qi J, Sun H, Zhang Y (2022). Single-cell and spatial analysis reveal interaction of FAP+ fibroblasts and SPP1+ macrophages in colorectal cancer. Nat Commun.

[56] Shen R, Li P, Zhang B (2022). Decoding the colorectal cancer ecosystem emphasizes the cooperative role of cancer cells, TAMs and CAFsin tumor progression. J Transl Med.

[57] Gok Yavuz B, Gunaydin G, Gedik ME (2019). Cancer associated fibroblasts sculpt tumour microenvironment by recruiting monocytes and inducing immunosuppressive PD-1+ TAMs. Sci Rep.

[58] Cho H, Seo Y, Loke KM (2018). Cancer-Stimulated CAFs Enhance Monocyte Differentiation and Protumoral TAM Activation via IL6 and GM-CSF Secretion. Clin Cancer Res.

[59] Olonisakin TF, Mayer A, Duvvuri U (2022). Cancer-associated Fibroblasts Transform Monocytes Into Protumorigenic Macrophages via IL-22 Signaling in Head and Neck Squamous Cell Carcinoma. Int J Radiat Oncol Biol Phys.

[60] Pakravan K, Mossahebi-Mohammadi M, Ghazimoradi MH (2022). Monocytes educated by cancer-associated fibroblasts secrete exosomal miR-181a to activate AKT signaling in breast cancer cells. J Transl Med.

[61] Wang Y-J, Fletcher R, Yu J (2018). Immunogenic effects of chemotherapy-induced tumor cell death. Genes Dis.

[62] Huntoon K, Lee D, Dong S (2023). Targeting phagocytosis to enhance antitumor immunity. Trends Cancer.

[63] Feng M, Jiang W, Kim BYS (2019). Phagocytosis checkpoints as new targets for cancer immunotherapy. *Nat Rev Cancer*.

[64] Koncina E, Nurmik M, Pozdeev VI (2023). IL1R1+ cancer-associated fibroblasts drive tumor development and immunosuppression in colorectal cancer. Nat Commun.

[65] Waldner MJ, Foersch S, Neurath MF (2012). Interleukin-6--a key regulator of colorectal cancer development. *Int J Biol Sci*.

[66] Zhong B, Cheng B, Huang X (2021). Colorectal cancer-associated fibroblasts promote metastasis by up-regulating LRG1 through stromal IL-6/STAT3 signaling. Cell Death Dis.

[67] Rossi J-F, Lu Z-Y, Jourdan M (2015). Interleukin-6 as a therapeutic target. Clin Cancer Res.

[68] Beyranvand Nejad E, Labrie C, van Elsas MJ (2021). IL-6 signaling in macrophages is required for immunotherapy-driven regression of tumors. J Immunother Cancer.

[69] Pascual-García M, Bonfill-Teixidor E, Planas-Rigol E (2019). LIF regulates CXCL9 in tumor-associated macrophages and prevents CD8+ T cell tumor-infiltration impairing anti-PD1 therapy. Nat Commun.

[70] Lai X, Dong Q, Xu F (2021). Correlation of c-MET expression with clinical characteristics and the prognosis of colorectal cancer. J Gastrointest Oncol.

[71] Feng X, Zhang Z, Sun P (2020). Interleukin-18 Is a Prognostic Marker and Plays a Tumor Suppressive Role in Colon Cancer. *Dis Markers*.

[72] Han ZQ, Assenberg M, Liu BL (2007). Development of a second-generation oncolytic Herpes simplex virus expressing TNFalpha for cancer therapy. *J Gene Med*.

[73] Hirvinen M, Rajecki M, Kapanen M (2015). Immunological effects of a tumor necrosis factor alpha-armed oncolytic adenovirus. *Hum Gene Ther*.

[74] Li W, Xu J, Zhao J (2017). Oxaliplatin and Infliximab Combination Synergizes in Inducing Colon Cancer Regression. *Med Sci Monit*.

[75] Stanilov N, Miteva L, Dobreva Z (2014). Colorectal cancer severity and survival in correlation with tumour necrosis factor-alpha. *Biotechnol Biotechnol Equip*.

[76] Qian Y, Yang T, Liang H (2022). Myeloid checkpoints for cancer immunotherapy. *Chin J Cancer Res*.

