# Supplementary material for: Cancer-associated fibroblasts shape early myeloid cell response to chemotherapy-induced immunogenic signals in next generation tumor organoid cultures
Source: J Immunother Cancer. 2024 Nov 4;12(11):e009494. doi: 10.1136/jitc-2024-009494 (PMC11535717; doi:10.1136/jitc-2024-009494)
Supplement: online supplemental figure 1 [file jitc-12-11-s001.pdf]

Supplementary Figures

Supplementary Figure 1.

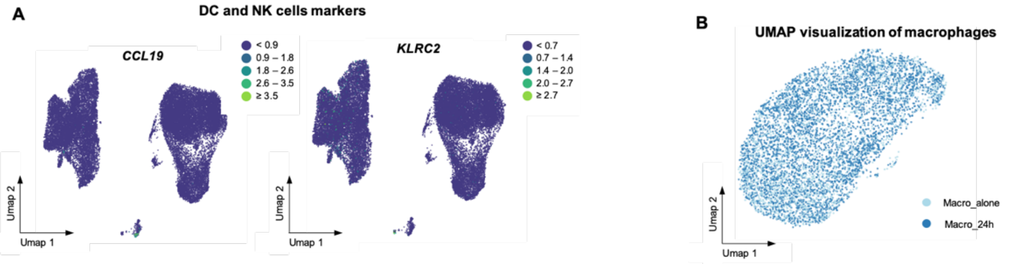

C GSEA of the Top 200 downregulated genes in monocytes co-cultured with CAFs for 24h (BioPlanet 2019)

Term	Adj P value	Top5 Overlap Genes
Lipid and lipoprotein metabolism	0.004383	*ABCC3, AGPAT5, PLA2G4C, CYP51A1, LPL*
Actin cytoskeleton regulatiom	0.004383	*MYLPF, TIAM1, MRAS, GSN, ITGA11*
Hemostatic pathway	0.0126	*CD84, DOCK3, DAGLA, SERPINE1, PDGFB*
Lipid digestion, mobilization, and transport	0.01318	*APOC2, LPL, APOE, A2M, MGLL*
TGF-beta regulation of extracellular matrix	0.01318	*EGR2, G6PD, SERPINE1, PLA2G4C, FN1*

D GSEA of the Top 25 upregulated genes in CAFs co-cultured with monocyts for 24h (MSigDB Hallmark 2020)

Term	Adj P value	Top5 Overlap Genes
TNF-α Signaling Via NF-κB	2.52E-06	*CXCL6, IL1B, CCL2, CXCL1, CXCL3*
Allograft Rejection	9.3E-04	*IL11, IL1B, CCL2, GBP2*
IL-6/JAK/STAT3 Signaling	1.02E-03	*IL1B, CXCL1, CXCL3*
Inflammatory Response	8.66E-03	*CXCL6, IL1B, CCL2*

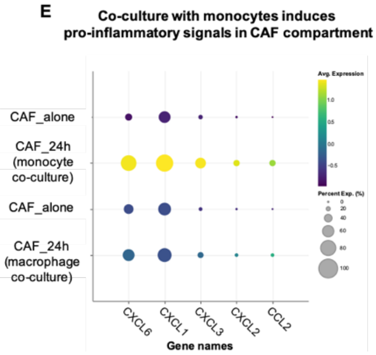

Supplementary Figure 1. A UMAP visualization of the co-cultured monocytic myeloid cells and CAF with overlaid expression of exemplary DC (CCL19) and NK cell (KLRC2) markers. **B** UMAP visualization of re-clustered macrophages from mono-culture (Macro_alone) and CAF co-culture (Macro_24h). **C** GSEA using Enrichr (BioPlanet 2019 database) of Top 200 downregulated genes in monocytes co-cultured 24h with CAF. **D** GSEA using Enrichr (MSigDB Hallmark 2020 database) of the Top 25 upregulated genes in CAF co-cultured for 24h with monocytes. **E** Dot plots representing average gene expression of pro-inflammatory cytokines in CAF in mono-cultures (CAF_alone) and co-cultures (CAF_24h from monocyte co-culture and CAF_24h from macrophage co-culture).

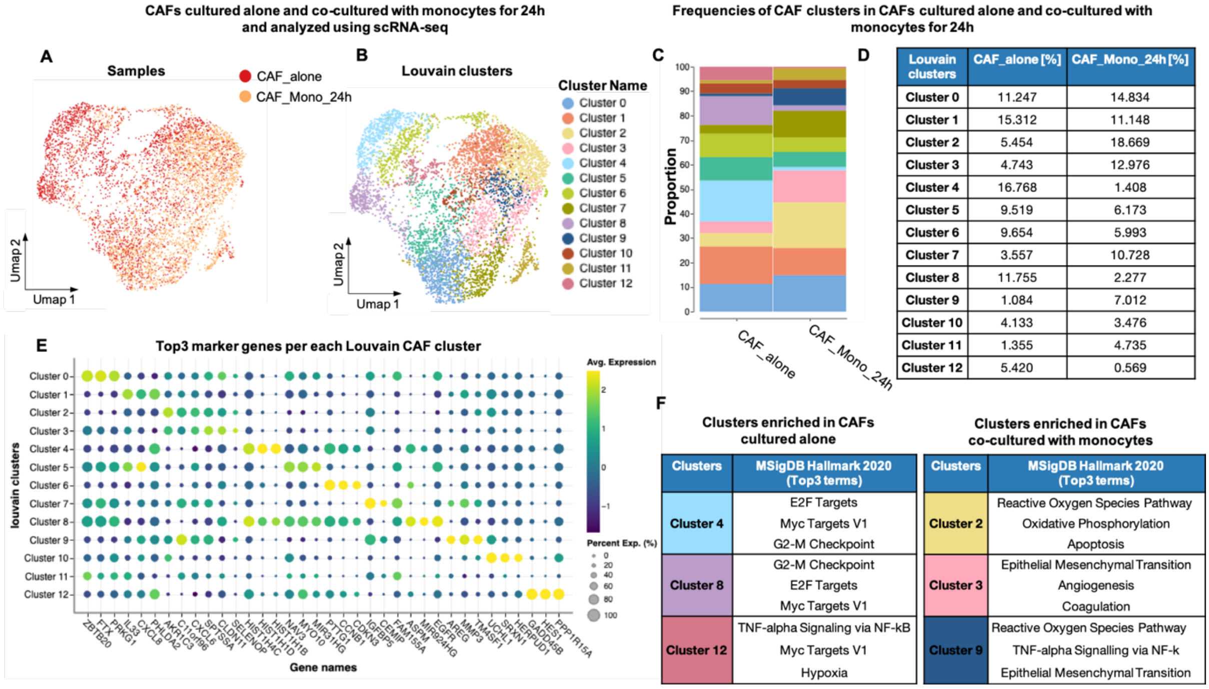

Louvain clusters	CAF_alone [%]	CAF_Mono_24h [%]
Cluster 0	11.247	14.834
Cluster 1	15.312	11.148
Cluster 2	5.454	18.669
Cluster 3	4.743	12.976
Cluster 4	16.768	1.408
Cluster 5	9.519	6.173
Cluster 6	9.654	5.993
Cluster 7	3.557	10.728
Cluster 8	11.755	2.277
Cluster 9	1.084	7.012
Cluster 10	4.133	3.476
Cluster 11	1.355	4.735
Cluster 12	5.420	0.569

Clusters enriched in CAFs cultured alone

Clusters	MSigDB Hallmark 2020 (Top3 terms)
Cluster 4	E2F Targets<br>Myc Targets V1<br>G2-M Checkpoint
Cluster 8	G2-M Checkpoint<br>E2F Targets<br>Myc Targets V1
Cluster 12	TNF-alpha Signaling via NF-kB<br>Myc Targets V1<br>Hypoxia

Clusters enriched in CAFs co-cultured with monocytes

Clusters	MSigDB Hallmark 2020 (Top3 terms)
Cluster 2	Reactive Oxygen Species Pathway<br>Oxidative Phosphorylation<br>Apoptosis
Cluster 3	Epithelial Mesenchymal Transition<br>Angiogenesis<br>Coagulation
Cluster 9	Reactive Oxygen Species Pathway<br>TNF-alpha Signalling via NF-k<br>Epithelial Mesenchymal Transition

Supplementary Figure 2. Sc-RNAseq analysis of re-clustered CAF cells cultured alone and co-cultured 24h with monocytes. A Visualization of re-clustered CAFs using UMAP projection based on the sample type. **B** Visualization of re-clustered CAFs using UMAP projection based on the Louvain clusters. **C** Frequency plot of Louvain clusters. **D** Table with detailed cluster frequencies depicted as % of all cells within the sample. **E** Dot plot showing average expressions of Top3 marker genes of each Louvain cluster. **F** Detailed characterization of enriched CAF clusters using GSEA analysis (Enrichr, MSigDB Hallmark 2020 database).

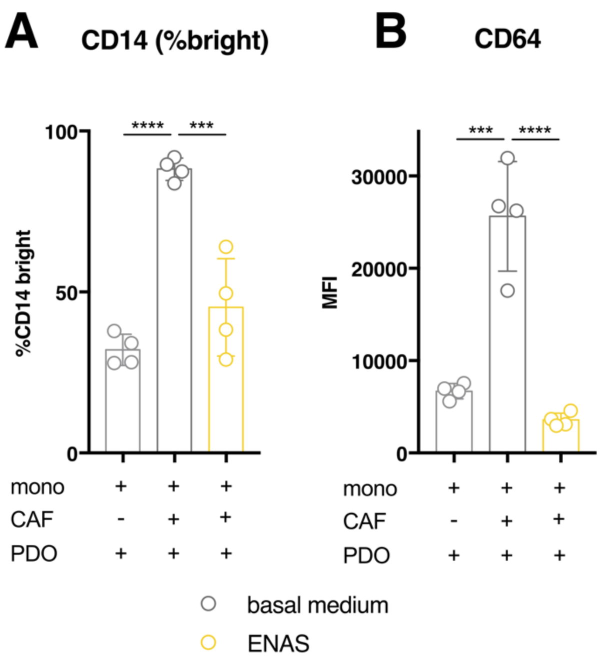

Supplementary Figure 3. A-B Color-coded scatter-plot circles represent flow cytometry measurements from individual monocyte donors. Presence of PDO, CAF and monocytes indicated below. **A** Quantification of $CD14^{+}$ cells. **B** Quantification of CD64 median fluorescence intensity.

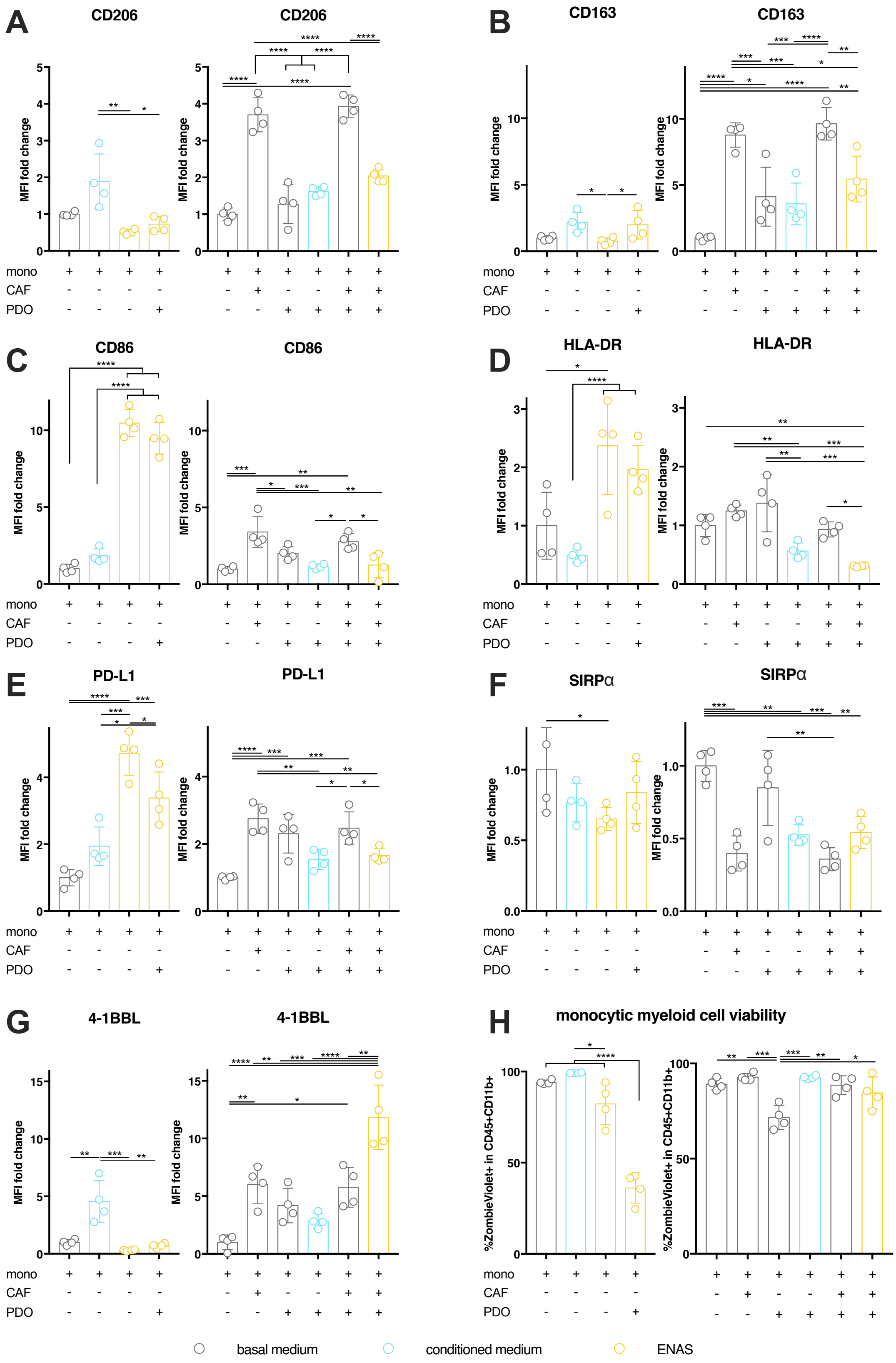

Supplementary Figure 4. Effect of various media conditions on monocyte and monocyte PDO cultures compared to complex cultures. A-H Quantification of **A** CD206, **B** CD163, **C** CD86, **D** HLA-DR, **E** PD-L1, **F** SIRPα, **G** 4-1BBL and **H** cell death in ECM submerged cultures after 72 hours via flow cytometry. The right respective panels represents additional controls, the

left panels represent normalized depiction of data shown in Figure 2E. Color-coded scatter-plot circles represent measurements from individual monocyte donors. Presence of PDO, CAF and monocytes indicated below.

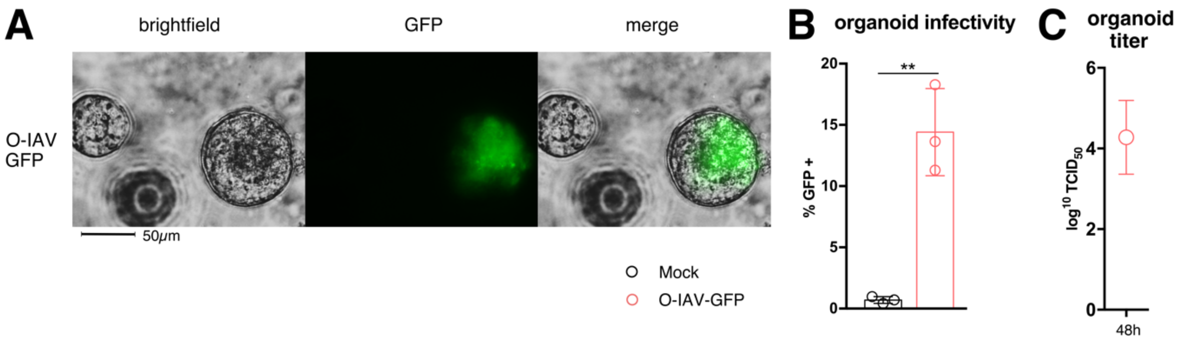

Supplementary Figure 5. A PDO in ECM were subjected to O-IAV-GFP infection. Microscopy after 24 hours. Brightfield and fluorescence channels indicated above, scale bar 50 μm. **B** PDO infected with O-IAV-GFP were released from ECM and dissociated to single cells. GFP+ cells were quantified via flow cytometry. **C** Supernatants of O-IAV-GFP infected PDO were harvested after 48 hours of culture and infectious titer determined via $TCID_{50}$.

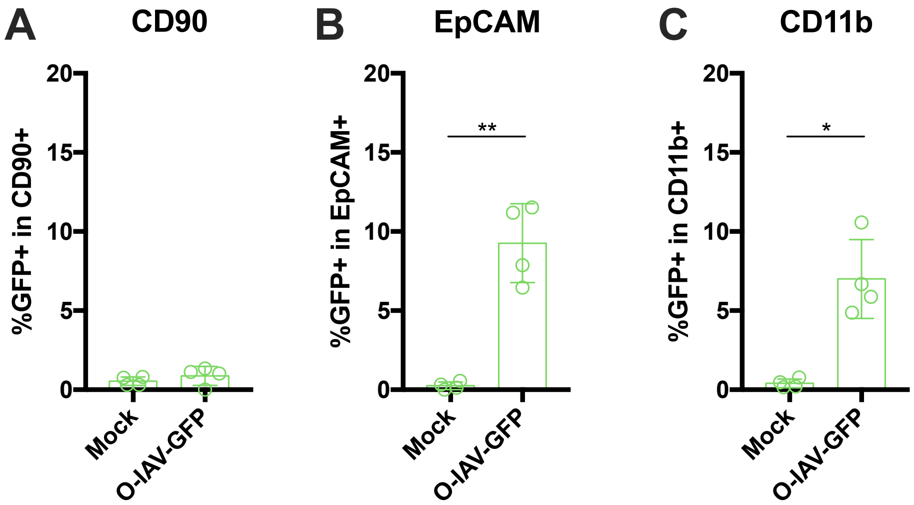

Supplementary Figure 6. Quantification of infected cells in triple cultures. Triple cultures consisting of PDO, CAF and monocytes were cultured for 72h, infected with the O-IAV-GFP virus and cultured for further 24h. Cells were stained for CD90 to indicate CAF, EpCAM to indicate PDO cells and CD11b to indicate cells of monocytic origin. Scatter plot circles represent measurements of GFP positive cells among **A** CD90 positive CAFs, **B** EpCAM positive PDO cells and **C** CD11b positive myeloid cells from separate experiments with individual monocyte donors. Viral infection or mock control is indicated below the column.

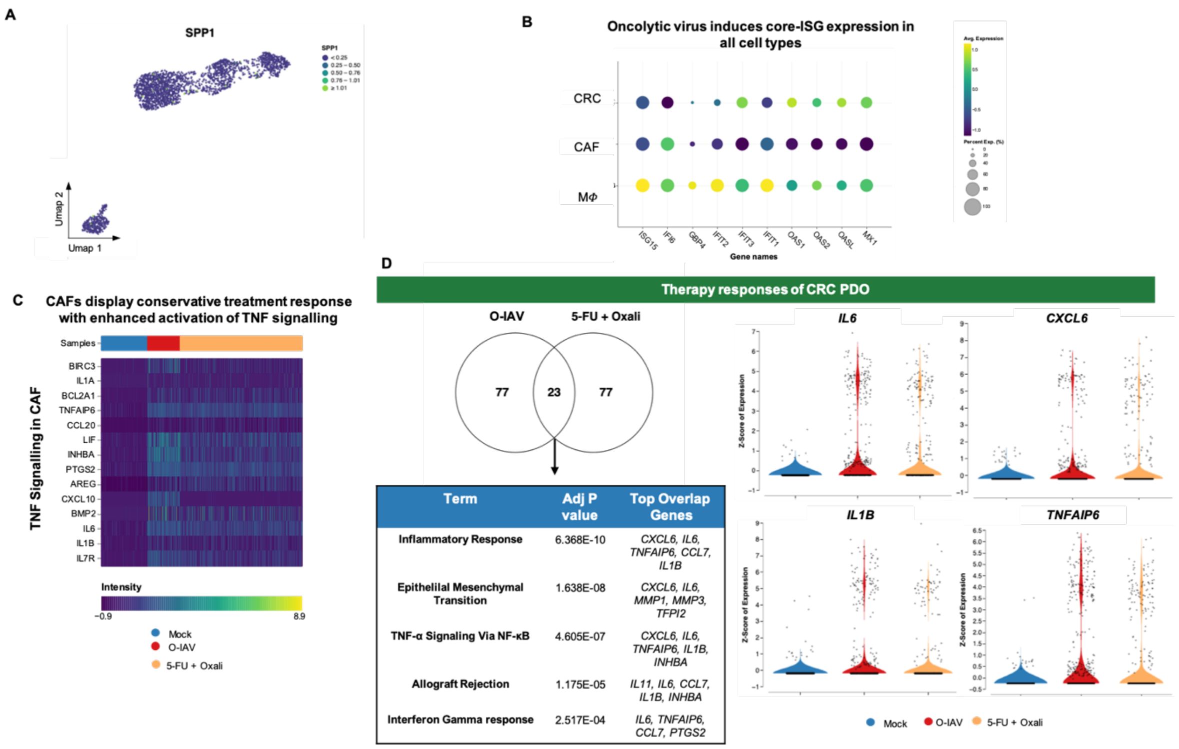

Term	Adj P value	Top Overlap Genes
Inflammatory Response	6.368E-10	*CXCL6, IL6, TNFAIP6, CCL7, IL1B*
Epithelilal Mesenchymal Transition	1.638E-08	*CXCL6, IL6, MMP1, MMP3, TFPI2*
TNF-α Signaling Via NF-κB	4.605E-07	*CXCL6, IL6, TNFAIP6, IL1B, INHBA*
Allograft Rejection	1.175E-05	*IL11, IL6, CCL7, IL1B, INHBA*
Interferon Gamma response	2.517E-04	*IL6, TNFAIP6, CCL7, PTGS2*

Supplementary Figure 7. A UMAP visualization of the re-clustered monocytic myeloid cells with overlaid expression of SPP1. **B** Dot plot visualization of the average expression of Type I and II IFN genes in all three cell types in the O-IAV treatment sample. **C** Heat-map of the Z-scored expression values of genes enriched in the TNF- α Signaling Via NF-κB pathway in GSEA (Enrichr, MSigDB Hallmark 2020 database) of the common treatment response signature in CAF. **D** Common treatment response signatures in CRC PDO cells visualized by Venn diagram (Venny 2.1), based on Top 100 upregulated genes in both treatments accompanied by the GSEA analysis (Enrichr, MSigDB Hallmark 2020 database, Table) of the common signature and violin plot visualization of expression of inflammatory genes from the common signature (*IL6*, *IL1B*, *CXCL6*, *TNFAIP6*).

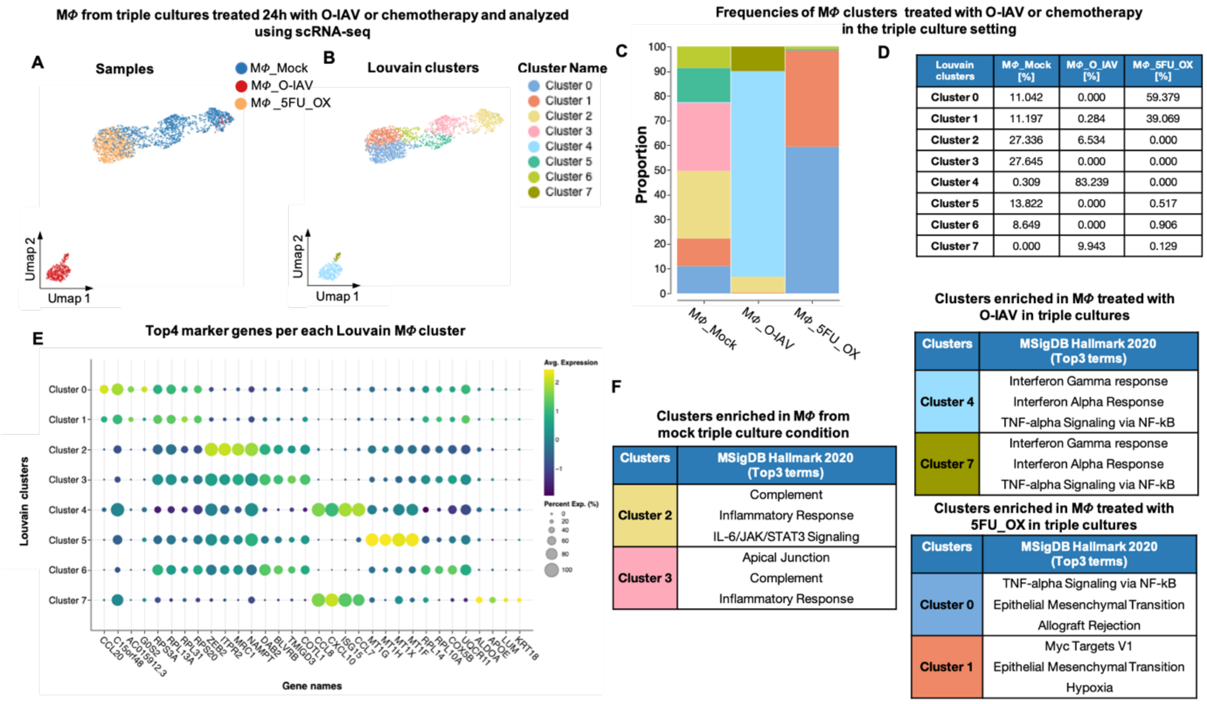

Supplementary Figure 8. Sc-RNAseq analysis of re-clustered TAM-like cells from the triple culture experiment. A Visualization of re-clustered TAM-like cells using UMAP projection based on the sample type. **B** Visualization of re-clustered TAM-like cells using UMAP projection based on the Louvain clusters. **C** Frequency plot of Louvain clusters. **D** Table with detailed cluster frequencies depicted as % of all cells within the sample. **E** Dot plot showing average expressions of Top4 marker genes of each Louvain cluster. **F** Detailed characterization of enriched TAM-like cell clusters using GSEA analysis (Enrichr, MSigDB Hallmark 2020 database).

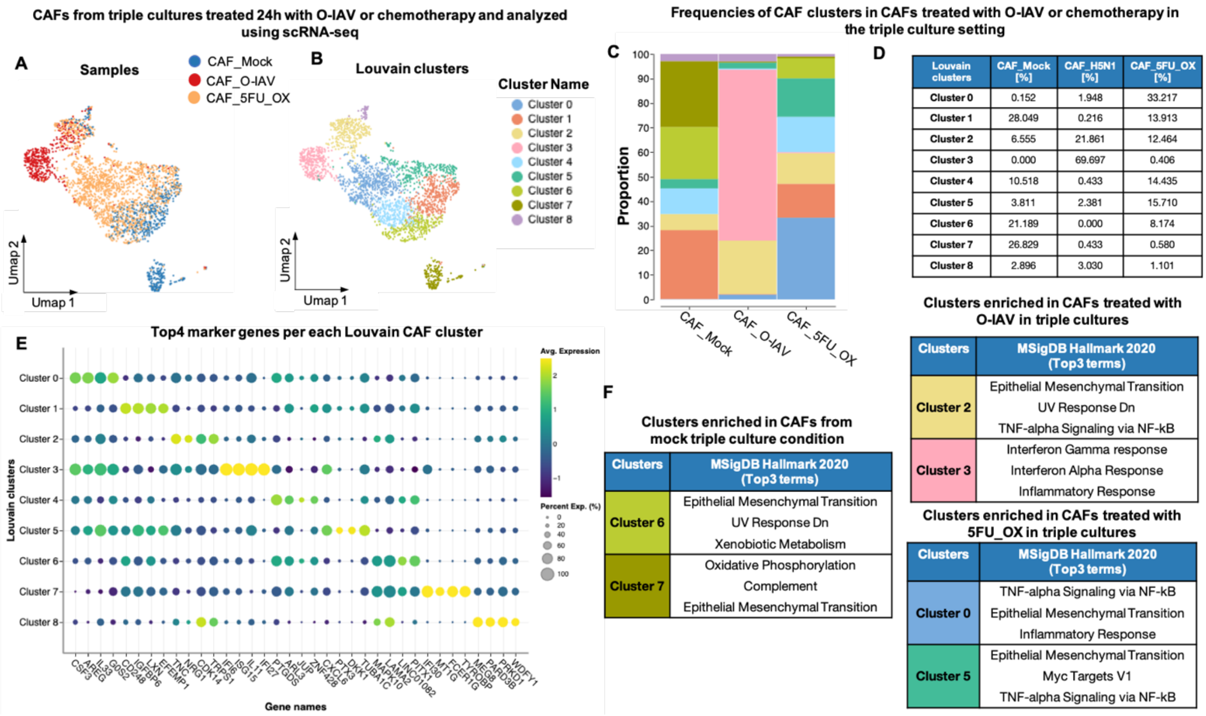

Supplementary Figure 9 Sc-RNAseq analysis of re-clustered CAFs from the triple culture experiment. A Visualization of re-clustered CAFs using UMAP projection based on the sample type. **B** Visualization of re-clustered CAFs using UMAP projection based on the Louvain clusters. **C** Frequency plot of Louvain clusters. **D** Table with detailed cluster frequencies depicted as % of all cells within the sample. **E** Dot plot showing average expressions of Top4 marker genes of each Louvain cluster. **F** Detailed characterization of enriched CAF clusters using GSEA analysis (Enrichr, MSigDB Hallmark 2020 database).

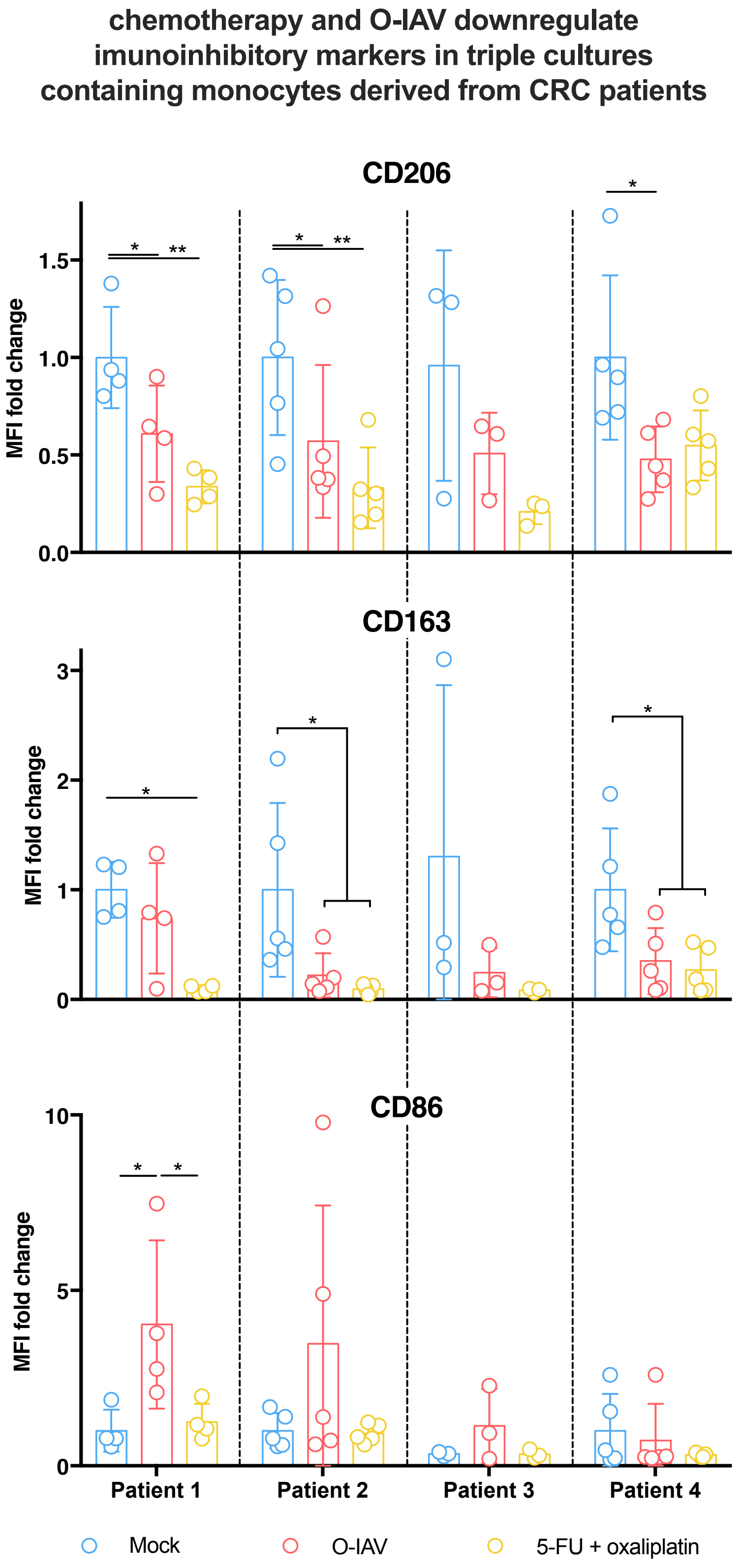

Supplementary Figure 10. Chemotherapeutics and oncolytic influenza A virus induce repolarization of TAM-like cells in primary triple culture assays performed with monocytes isolated from CRC patients. Quantification of CD206, CD163 and CD86 median fluorescence intensity (MFI) normalized to the corresponding patient's MFI from untreated condition, measured on TAM-like cells from treated triple cultures.

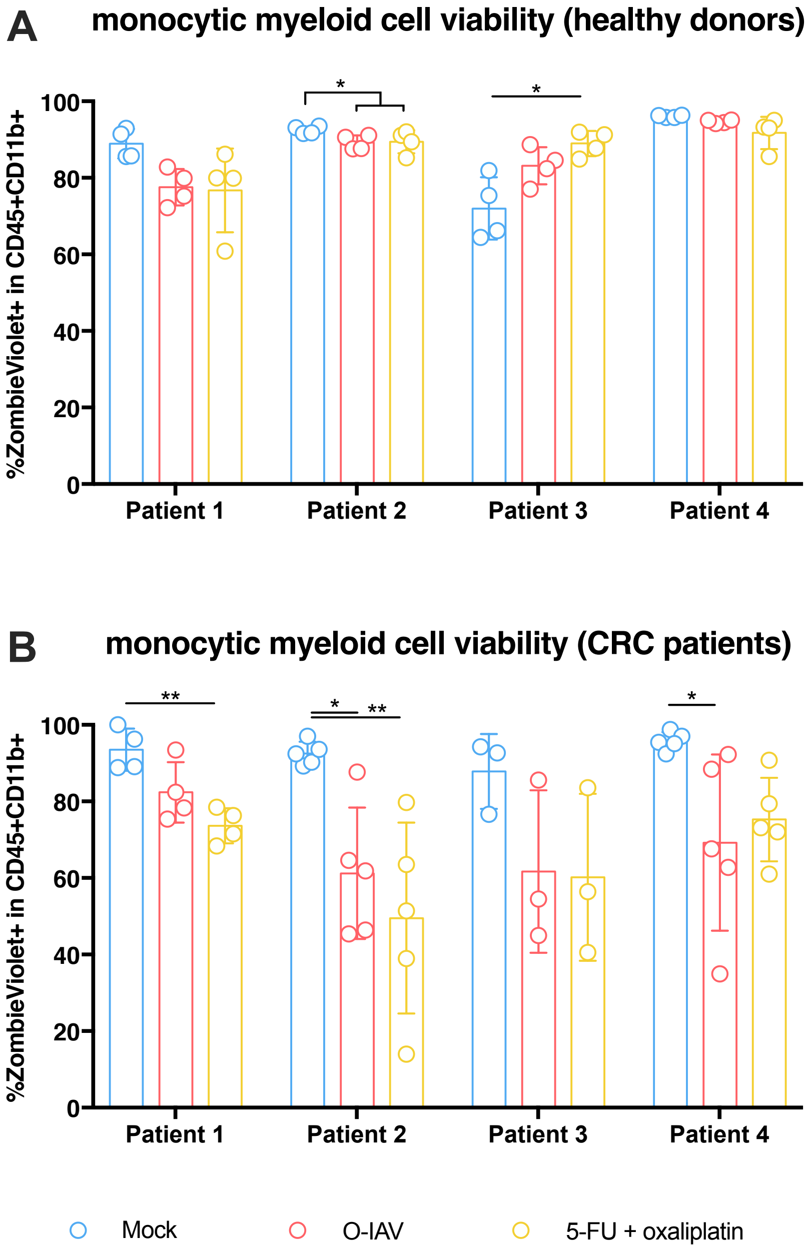

Supplementary Figure 11. TAM-like monocytic myeloid cell viability in chemotherapy and O-IAV treated co-cultures. A Viability of TAM-like cells in triple cultures containing healthy donor monocytes treated with chemotherapeutics or O-IAV was quantified via flow cytometry. **B** Viability of TAM-like cells in triple cultures containing CRC patient monocytes treated with chemotherapeutics or O-IAV was quantified via flow cytometry.

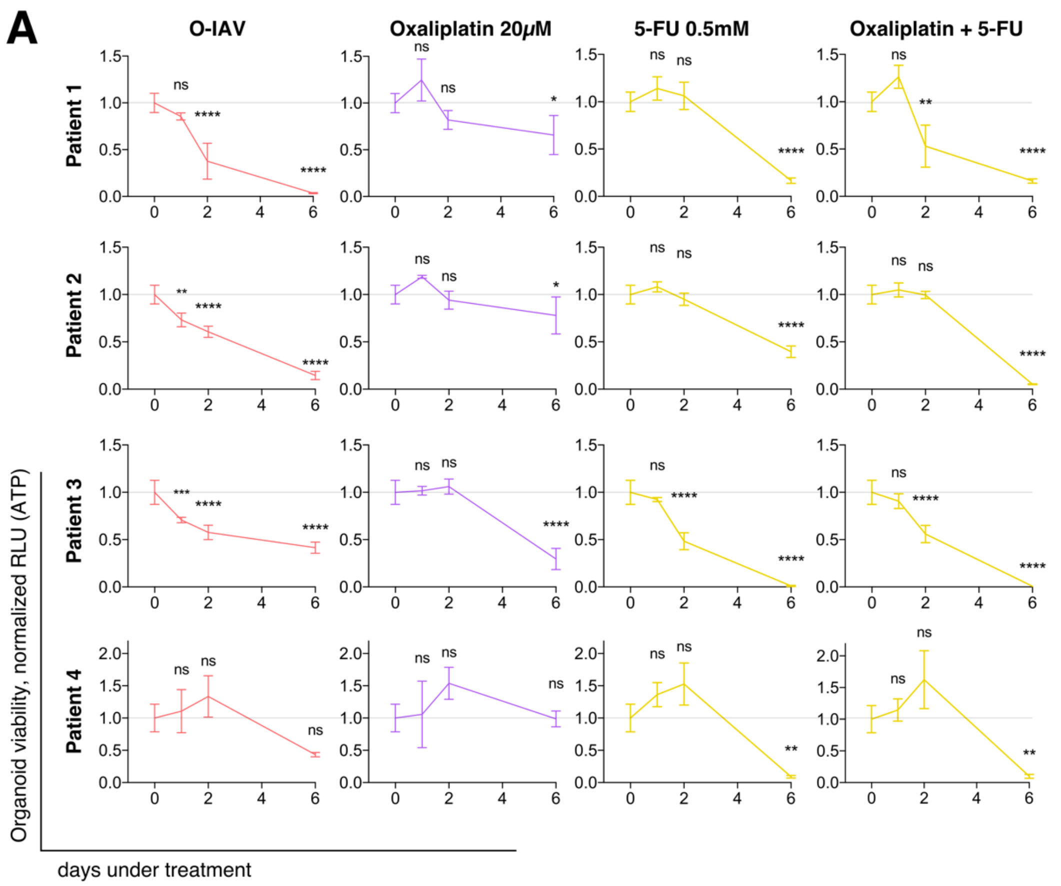

Supplementary Figure 12. A PDO were subjected to the indicated treatments for the indicated times. Cell viability was determined via CellTiterGlo© assay.

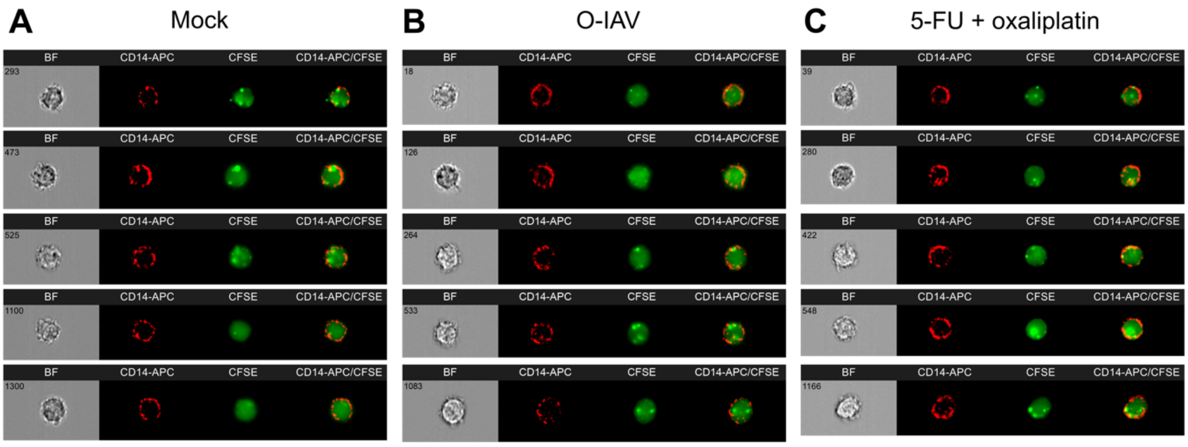

Supplementary Figure 13. A-C PDO from patient 3 were seeded in ECM domes and subjected to **A** no treatment for 6 days, **B** no treatment until O-IAV infection on day 5 after seeding and one further day of incubation or **C** 5-FU and oxaliplatin treatment for 6 days. Phagocytosis assay was performed with healthy donor monocytes and cells stained with an APC labeled CD14 antibody and ZombieViolet™. The cells were then subjected to readout via imaging-flow-cytometry. Five representative events are shown per treatment. Each panel corresponding to a single event contains a brightfield (BF) image with the event number indicated in the top left corner, a CD14-APC image, a CFSE image and an overlay of CD14-APC and CFSE. All putative phagocytotic events, gated here as CFSE/CD14 double positive, appeared as CFSE signal fully engulfed by CD14 signal, strongly indicating proper phagocytosis as opposed to adherence.
